# Ordered Mesopore Confined Pt Nanoclusters Enable Unusual Self-Enhancing Catalysis

**DOI:** 10.1021/acscentsci.2c01290

**Published:** 2022-12-16

**Authors:** Meiqi Gao, Zhirong Yang, Haijiao Zhang, Junhao Ma, Yidong Zou, Xiaowei Cheng, Limin Wu, Dongyuan Zhao, Yonghui Deng

**Affiliations:** † Department of Chemistry, Department of Gastroenterology and Hepatology, Zhongshan Hospital, State Key Laboratory of Molecular Engineering of Polymers, Shanghai Key Laboratory of Molecular Catalysis and Innovative Materials, Collaborative Innovation Center of Chemistry for Energy Materials (iChEM), Fudan University, Shanghai200433, China; ‡ State Key Laboratory of Chemical Engineering, East China University of Science and Technology, Shanghai200237, China; § Institute of Nanochemistry and Nanobiology, School of Environmental and Chemical Engineering, Shanghai University, Shanghai200444, People’s Republic of China; ∥ Institute of Energy and Materials Chemistry, Inner Mongolia University, Hohhot010021, China

## Abstract

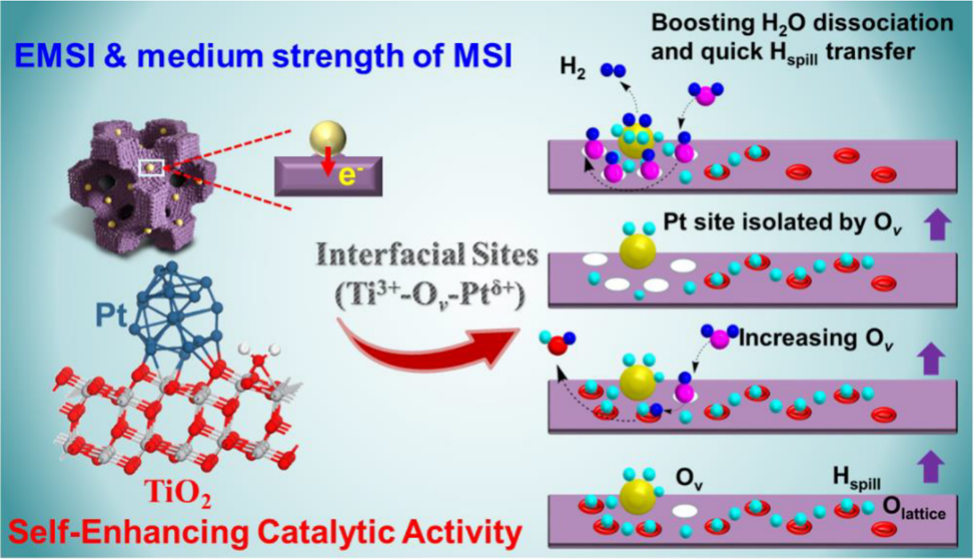

As an important kind of emerging heterogeneous catalyst for sustainable chemical processes, supported metal cluster (SMC) catalysts have received great attention for their outstanding activity; however, the easy aggregation of metal clusters due to their migration along the substrate’s surface usually deteriorates their activity and even causes catalyst failure during cycling. Herein, stable Pt nanoclusters (NCs, ∼1.06 nm) are homogeneously confined in the uniform spherical mesopores of mesoporous titania (mpTiO_2_) by the interaction between Pt NCs and metal oxide pore walls made of polycrystalline anatase TiO_2_. The obtained Pt-mpTiO_2_ exhibits excellent stability with well-retained CO conversion (∼95.0%) and Pt NCs (∼1.20 nm) in the long term water–gas shift (WGS) reaction. More importantly, the Pt-mpTiO_2_ displays an unusual increasing activity during the cyclic catalyzing WGS reaction, which was found to stem from the *in situ* generation of interfacial active sites (Ti^3+^-O_v_-Pt^δ+^) by the reduction effect of spillover hydrogen generated at the stably supported Pt NCs. The Pt-mpTiO_2_ catalysts also show superior performance toward the selective hydrogenation of furfural to 2-methylfuran. This work discloses an efficient and robust Pt-mpTiO_2_ catalyst and systematically elucidates the mechanism underlying its unique catalytic activity, which helps to design stable SMC catalysts with self-enhancing interfacial activity in sustainable heterogeneous catalysis.

## Abstract 2

Deposition of Pt nanoclusters in nanopores of mesoporous TiO_2_ can confine Pt species in the pore wall and *in situ* generates numerous interfacial active sites (Ti^3+^-O_v_-Pt^δ+^) during reaction to improve cycling WGS catalytic activity.

## Introduction

The heterogeneous catalytic reaction is an important sustainable chemical process in industry production, and such a process is strongly susceptible to the surface and interface properties (e.g., acidity/basicity, active sites, coordination states) and micro/nano structure (e.g., size, facets, defects) of catalysts.^1−4^ Tailoring the property of nanosized metals at the interfacial sites of heterogeneous catalysts is crucial to forming excellent active sites and improving the catalyst efficiency.^4−7^ Thereby, the interfacial sites induced by metal–support interaction (MSI) play a decisive role in most heterogeneous catalytic processes, such as CO oxidation,^8,9^ methanol oxidation,^10^ CO_2_ reduction,^11−13^ and the water–gas shift (WGS) reaction.^14−16^ In terms of the WGS reaction, which is vital for eliminating carbon monoxide and solving the energy crisis, the reaction between carbon monoxide and water to produce clean energy hydrogen is strongly dependent on the interfacial sites and active species.

Recently, considerable attention has been paid to building strong metal–support interaction (SMSI) systems using reducible metal oxide supports, aiming to develop stable heterogeneous catalysts.^17−23^ However, SMSI can lead to overencapsulation of metal nanoparticles (NPs) by the reducible metal oxide supports, especially for large metal NPs of several nanometers, eventually impeding the exposure of interfacial active sites to achieve high catalytic efficiency.^24^ Suppression of the size of metal nanoparticles at the support’s surface was demonstrated to be a reliable approach to realizing an appropriate encapsulation of metal NPs and generate a favorable metal/support interface.^25−31^ Such a metal/support interface was originally found in CeO_2_ particle-supported Pt catalyst^27^ and later defined as the electronic metal–support interaction (EMSI).^28^ The EMSI can induce a strong electronic perturbation between ultrafine metal nanoclusters of less than 2 nm and reducible metal oxides, contributing to excellent catalytic activity.^29−31^ Ma and co-workers have fabricated series of stable supported metal cluster (SMC) catalysts and clarified their advantages in various important catalytic fields.^32−35^ Designed construction of an appropriate MSI is extremely important to creating SMC catalysts with high stability and activity.^36^ The stable interfacial sites with an appropriate MSI allow for excellent performance toward the WGS reaction that is widely used in the chemical industry (e.g., production and purification of H_2_) due to the accelerated H_2_O dissociation and intermediate transfer.^37−43^

To date, various methods have been reported for the synthesis of SMC catalysts using nonporous or porous supports.^31,44,45^ Nonporous supports cannot guarantee the long-term stability of SMC catalysts because of the inevitable aggregation of metal NCs especially at high working temperatures.^46^ Microporous materials (pore size <2 nm) including zeolites can exert a nanoconfinement effect for NCs to ensure the stability by preventing aggregation;^47−50^ however, the resultant SMC catalysts mainly provide dominant surface diffusion of reactant molecules during heterogeneous catalysis, which is unfavorable for catalysis due to the limited accessibility of active sites. By contrast, mesoporous materials have larger uniform nanopores of 2–50 nm, which are comparable to the free mean paths of gaseous molecules, and tunable pore wall microenvironments (e.g., active sites, surface acidity, surface reducibility).^51−53^ The highly interconnected mesopores facilitate the transportation of gas molecules following Knudsen diffusion within the mesoporous matrix,^54−56^ which is particularly favorable for the guest molecules efficiently interacting with the pore wall to achieve improved catalytic activity.^57,58^

In this work, a highly efficient and stable SMC catalyst was constructed by confinement of ultrafine Pt NCs in the mesopores of mesoporous titania (mpTiO_2_). The resultant Pt-mpTiO_2_ catalysts possess well dispersed Pt NCs and a favorable EMSI effect and exhibited an excellent long-term stability in catalyzing the WGS reaction due to the well retained metal/metal oxide interfaces in the mesoporous matrix during reaction. Strikingly, the Pt-mpTiO_2_ catalysts exhibited an unexpected increasing activity during repeated catalyzing of the WGS reaction. Such a self-enhancement phenomenon was found to stem from the continuous formation of Ti^3+^-O_v_-Pt^δ+^ active sites at the interfaces of the confined Pt NCs and the TiO_2_ pore wall. These novel Pt-mpTiO_2_ catalysts were demonstrated to be efficient not only for the WGS reaction but also for other catalytic reactions such as hydrogenation and the photocatalytic reaction. These findings provide new insights for development of nanostructured materials with tailored metal–metal oxide interfacial microenvironments and superior activities.

## Results and Discussion

**Figure 1:**
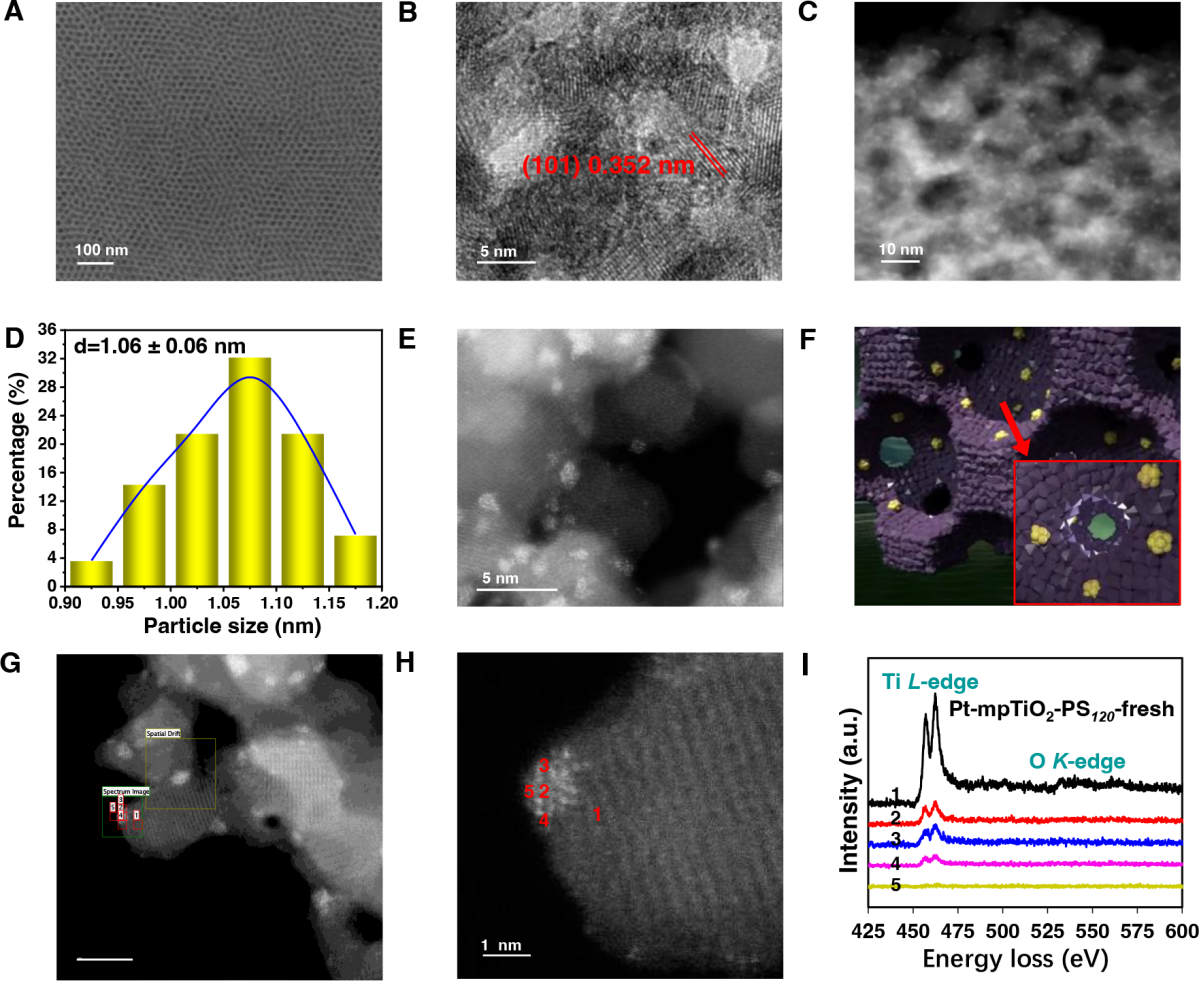
Structure analysis of the Pt-mpTiO_2_-PS_120_. (A) FESEM, (B) HRTEM, (C) HAADF-STEM images of Pt-mpTiO_2_-PS_120_. (D) Particle size distribution of Pt on Pt-mpTiO_2_-PS_120_ obtained according to the HAADF-STEM image. (E) Ac-HAADF-STEM image of Pt-mpTiO_2_-PS_120_. (F) Schematic illustration of the internal structure of Pt-mpTiO_2_-PS_120_. (Yellow, Pt NCs; purple, TiO_2_ crystal particle.) (G–I) EELS analysis of Pt-mpTiO_2_-PS_120_. (G) Survey image for the EELS analysis, scale: 5 nm. (H) The corresponding Ac-HAADF-STEM image. (I) The EELS spectra of the selected spots (red mark) in G and H.

It started from the synthesis of mesoporous TiO_2_ (mpTiO_2_-PS_*x*_, *x* refers to the repeating unit number of styrene: 120, 173, or 248) supports via the solvent evaporation induced coassembly (EICA) approach (Scheme S1), where lab-made amphiphilic poly(ethyl oxide)-*block*-polystyrene (PEO-*b*-PS_*x*_) diblock copolymers and tetrabutyl orthotitanate (TBOT) were used as the template (the porogen) and titania precursor, respectively. During the assembly process, the hydrophobic PS segments can aggregate as a micellar inner core, and the hydrophilic PEO segments interact with hydrolyzed titanium precursor during the solvent evaporation induced coassembly process. In addition, the pore size of mesoporous TiO_2_ can be flexibly regulated by changing the molecular weight of PS segments. Thanks to the unique structure-directing effect of the PEO-*b*-PS_*x*_ template, the obtained mpTiO_2_ has interconnected uniform spherical mesopores (pore size 11.2, 14.9, and 24.6 nm, respectively), a high specific surface area (140 m^2^/g, 113 m^2^/g, and 92.3 m^2^/g, respectively), and a polycrystalline pore wall made of anatase TiO_2_ nanocrystals (Figures S1 and S2 and Table S1). For comparison, nonporous anatase TiO_2_ (npTiO_2_) was also synthesized via a similar procedure without adding PEO-*b*-PS_*x*_. The npTiO_2_ powder (Figure S3) has irregular structure with a low specific surface area (2.94 m^2^/g) due to the uncontrolled sintering and aggregation of TiO_2_ NPs without the structure-directing effect of the PEO-*b*-PS_*x*_ template.^59,60^ After loading Pt NCs into the mesopores using chloroplatinic acid hexahydrate as a precursor, novel SMC catalysts can be obtained, and the ordered mesoporous structure is well-retained for the Pt-mpTiO_2_-PS_120_, as confirmed by electron microscopy observation (Figure 1A–C and Figure S4), N_2_ adsorption–desorption isotherms (Figure S1 and Table S1), X-ray diffraction (XRD) (Figure S5), and small-angle X-ray scattering (SAXS) measurements (Figure S1). The thermogravimetric analysis (TGA) result shows that the mpTiO_2_ obtained after calcination in air at 450 °C for 30 min has no visible weight loss at temperatures above 450 °C in the air (Figure S6), and it confirms that residual carbon has been completely removed after calcination in the air. Notably, this facile in-pore deposition method allows for a convenient synthesis of Pt-mpTiO_2_ catalysts with a high loading amount of Pt NCs up to 1.72 wt % (Table S2), and the obtained ultrafine Pt NCs with a size of 1.06 ± 0.06 nm (Figure 1D) are homogenesouly distributed in the mesopores of the mpTiO_2_, due to the high specific surface area and favorable confinement effect of mpTiO_2_-PS_120_ (Figures 1E,F). Aberration-corrected high angle annular dark-field scanning transmission electron microscopy (ac-HAADF-STEM) images (Figure 1E) reveal that the Pt NCs in Pt-mpTiO_2_-PS_120_ consist of many Pt atoms. The formation of such a unique nanocluster structure of Pt is possibly due to the hydrophilic rough pore wall of mpTiO_2_ formed by TiO_2_ nanocrystals, which helps to spread the Pt precursor solution but prevent Pt species from aggregation during reduction.^61^ By contrast, much larger Pt particles of 3.38 ± 0.72 and 3.25 ± 0.73 nm, respectively, were obtained when the as-synthesized npTiO_2_ and commercial anatase TiO_2_ (comTiO_2_) powders were used as the supports (Figures S3 and S7). These results clearly indicate that mpTiO_2_ as the supports have unique advantage for preparing SMC catalysts with highly dispersed Pt NCs. Moreover, the electron energy loss spectrum (EELS) of Pt-mpTiO_2_-PS_120_ collected at spots 1, 2, 3, 4, and 5 (Figure 1G–I) shows that signals of the Ti *L*-edge and O *K*-edge were observed at spots 2, 3, and 4, similar to those at spot 1, while no Ti *L*-edge and O *K*-edge signals were observed at spot 5. This result demonstrates a partial encapsulation of Pt NCs by mpTiO_2_-PS_120_, indicating a favorable medium MSI for Pt-mpTiO_2_-PS_120_.

**Figure 2:**
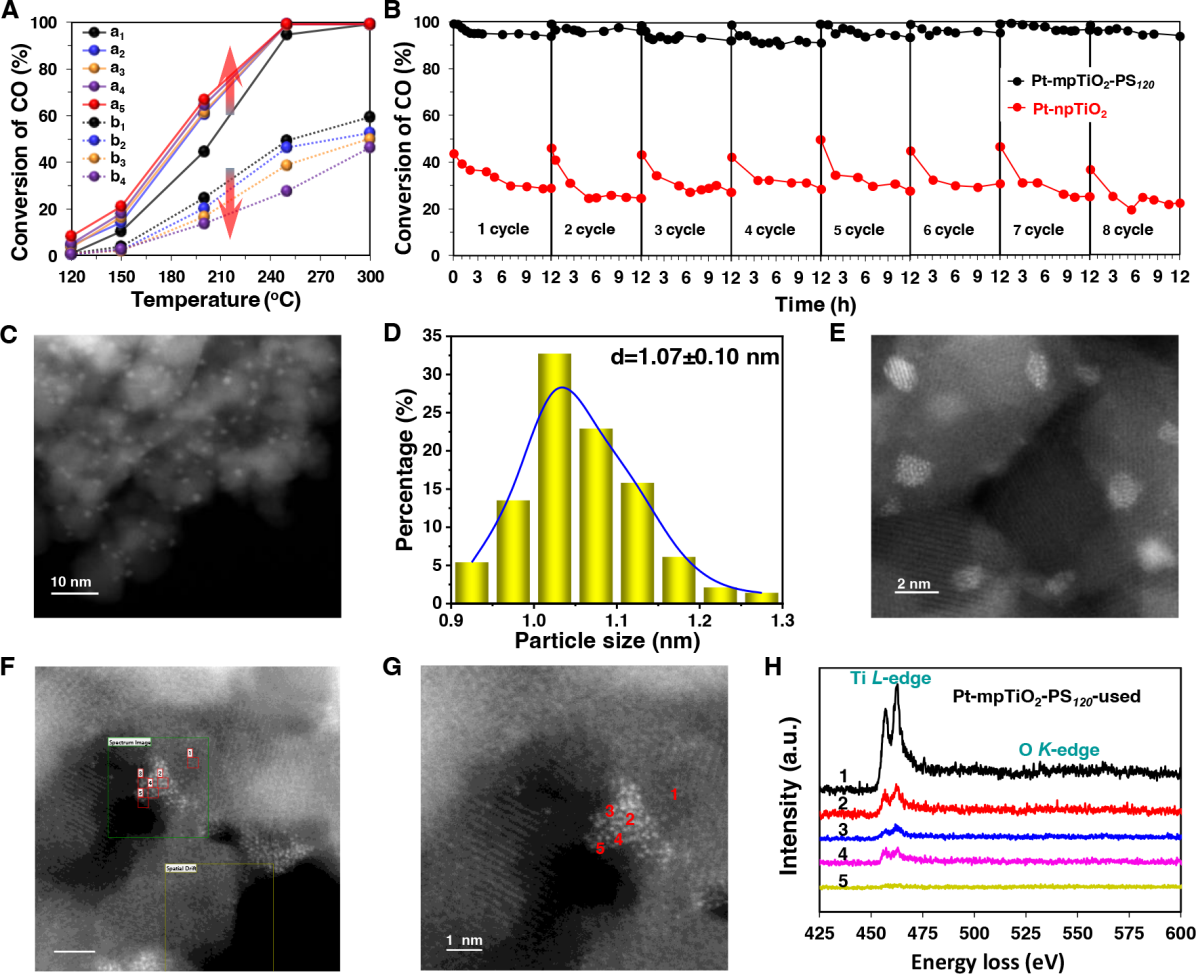
Catalytic performance of Pt-mpTiO_2_-PS_120_ towards the water–gas shift reaction using Pt-npTiO_2_ as a contrast and the structure analysis of used Pt-mpTiO_2_-PS_120_. (A) CO conversion curves as a function of temperature over the (a) Pt-mpTiO_2_-PS_120_ and (b) Pt-npTiO_2_ during the cyclic catalytic activity evaluation five times (reaction conditions: 2% CO, 8% H_2_O, 90% N_2_; WHSV 60 000 mL g_cat_^–1^ h^–1^). (B) Plots of CO conversion to CO_2_ as a function of reaction time at 250 °C over the Pt-mpTiO_2_-PS_120_ and Pt-npTiO_2_ for the catalytic stability evaluation. (C) HAADF-STEM image of used Pt-mpTiO_2_-PS_120_ after cyclic activity evaluation five times. (D) Particle size distribution of Pt NCs on used Pt-mpTiO_2_-PS_120_ after cyclic activity evaluation five times obtained according to the HAADF-STEM image. (E) Ac-HAADF-STEM image of used Pt-mpTiO_2_-PS_120_ after cyclic activity evaluation five times. (F–H) EELS analysis of used Pt-mpTiO_2_-PS_120_ after cyclic catalytic activity evaluation five times. (F) Survey image for the EELS analysis, scale: 2 nm. (G) The corresponding Ac-HAADF-STEM image. (H) The EELS spectra of the selected spots (red mark) in F and G.

The WGS reaction was used as a model to investigate the catalytic behavior of these Pt-TiO_2_ catalysts (Figure 2 and Figures S8 and S9), which represents an important energy conversion technology in the chemical industry. The Pt-npTiO_2_ shows a poor catalytic activity with a CO conversion of 49.5% at 250 °C (Figure 2A, b_1_). In contrast, the Pt-mpTiO_2_-PS_120_ exhibits excellent catalytic activity, and the CO conversion reaches 94.8% at 250 °C (Figure 2A, a_1_). Remarkably, different from the continuously decreased activity of the used Pt-npTiO_2_ during the cycling test (Figure 2A, b_1_–b_4_), the activity of used Pt-mpTiO_2_-PS_120_ increases significantly with a CO conversion of 99.1% at 250 °C as a contrast to the fresh one, indicating an unusual self-enhancement of catalytic activity (Figure 2A, a_1_–a_5_). Such an unexpected behavior is dramatically different from conventional catalysts whose activities usually decrease during the cycling test. Moreover, in comparison with Pt-npTiO_2_, Pt-mpTiO_2_-PS_120_ exhibits well-maintained CO conversion (∼95.0%) at 250 °C within eight cycles for about 96 h (Figure 2B). After the WGS reaction, the mesoporous structure (Figure 2C and Figures S10 and S11) and crystal phase (Figure S12) of Pt-mpTiO_2_-PS_120_ are well preserved, indicating good stability. The high dispersion state of Pt NCs was well preserved (Figure 2C,E), and the mean diameter is 1.07 ± 0.10 nm (Figure 2D), very close to that (∼1.06 nm) of fresh Pt-mpTiO_2_-PS_120_. According to the EELS spectroscopy of the used Pt-mpTiO_2_-PS_120_ collected at spots 1, 2, 3, 4, and 5 (Figure 2F–H), a partially encapsulated structure of Pt NC by mpTiO_2_-PS_120_ is also found, indicating a well-retained medium encapsulation state of Pt NCs. All of these results clearly demonstrate an excellent stability of Pt-mpTiO_2_-PS_120_ due to the preservation of medium MSI between Pt NCs and mpTiO_2_ support.

**Figure 3:**
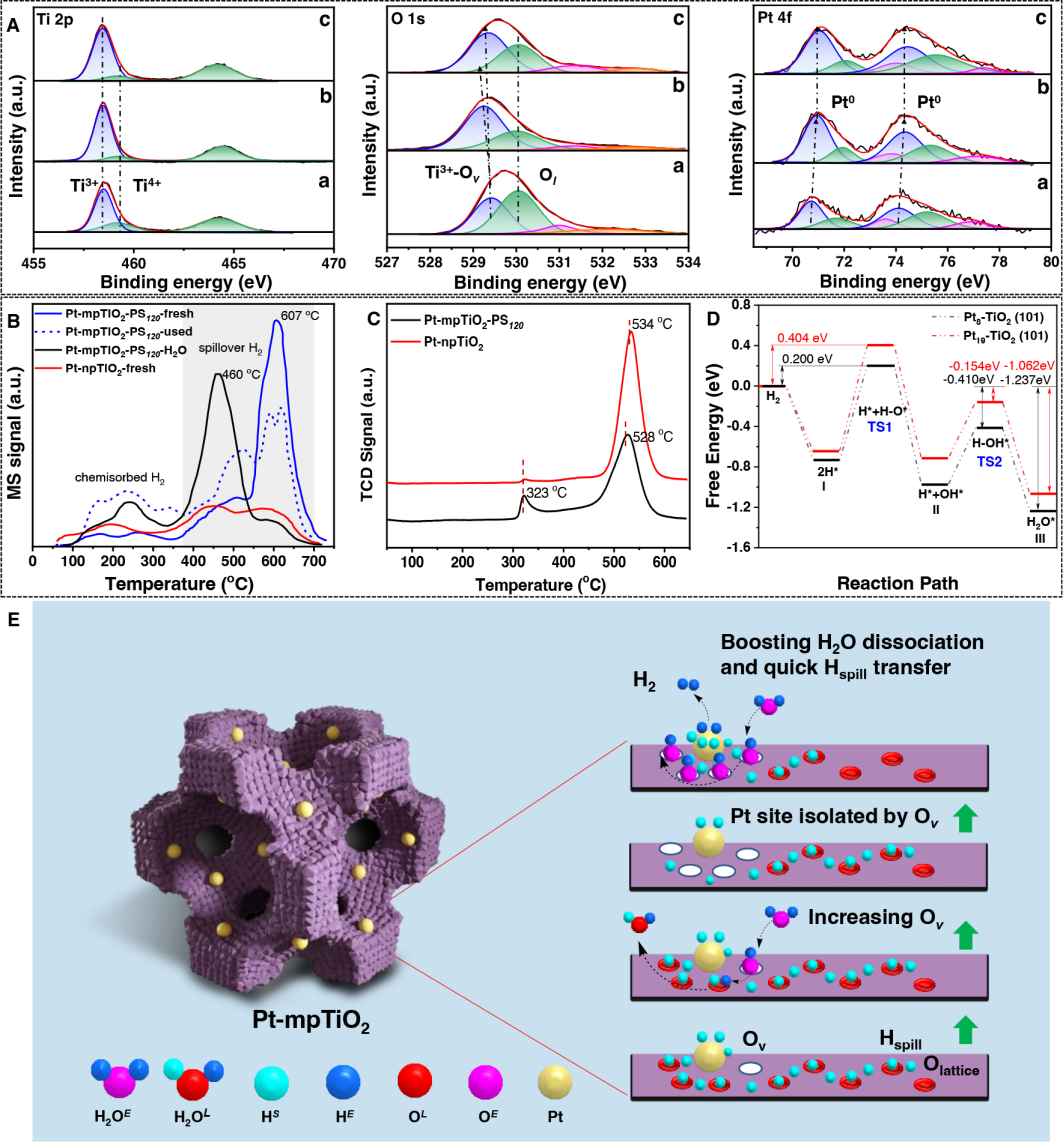
Proposed mechanism for the unexpected enhanced cyclic catalytic ability for Pt-mpTiO_2_-PS_120_. (A) XPS spectra of Pt 4f, Ti 2p, and O 1s of the (a) fresh Pt-mpTiO_2_-PS_120_, (b) used Pt-mpTiO_2_-PS_120_ after cyclic activity evaluation five times, and (c) used Pt-mpTiO_2_-PS_120_ after catalytic stability and cyclic performance evaluation for about 96 h at 250 °C. (B) H_2_-TPD profiles of the fresh Pt-mpTiO_2_-PS_120_ and Pt-npTiO_2_, used Pt-mpTiO_2_-PS_120_ after cyclic activity evaluation five times, as well as Pt-mpTiO_2_-PS_120_ treated in a H_2_O atmosphere. The signals at higher temperature in the gray area belong to the desorption peak of spillover hydrogen. (C) H_2_-TPR profiles of fresh Pt-mpTiO_2_-PS_120_ and Pt-npTiO_2_. (D) The calculated free energy for the reaction path of O_v_ formation on Pt_8_–TiO_2_ (101) and Pt_19_–TiO_2_ (101) by hydrogen reduction. (E) Scheme of the mechanism and effect of O_v_ increase in used Pt-mpTiO_2_-PS_120_. First, lattice oxygen (O^*L*^) on mpTiO_2_ is activated by the hydrogen radical (H^*E*^) derived from the dissociation of H_2_O (H_2_O^*E*^) at the adjacent O_v_. Then, the concentration of O_v_ increases combined with the release of H_2_O (H_2_O^*L*^) molecules due to the hydrogen (H^*S*^) spillover effect cooperating with the activation of lattice oxygen. As a result of the increased O_v_, dissociation of H_2_O^*E*^ and stabilization of Pt NCs are promoted, and the hydrogen (H^*E*^_2_) desorption is in turn accelerated due to the existence of hydroxyl (O^*E*^H^*E*^) derived from H_2_O (H_2_O^*E*^) dissociation.

X-ray photoelectron spectroscopy (XPS) measurements reveal that the used catalyst has a higher metallic Pt (Pt^0^/ Pt^0^ + Pt^2+^ + Pt^4+^) ratio (0.56) than the fresh one (0.47) due to the reduction of *in situ* generated H_2_ during the WGS reaction (Figure 3A and Table S3). Moreover, the Pt NCs in the used catalyst after a long-term WGS reaction for about 96 h only slightly increase to 1.20 ± 0.20 nm (Figure S11), indicating a good long-term stability of the Pt-mpTiO_2_-PS_120_ catalyst. Such firmly supported Pt NCs are beneficial to maintaining the EMSI between Pt NCs and mpTiO_2_. It is worth noting that the obtained Pt-mpTiO_2_-PS_120_ catalyst exhibits outstanding catalytic activity of up to 13.5 times higher than previously reported similar catalysts working at the same temperature (Table S4). Similarly, both Pt-mpTiO_2_-PS_173_ and Pt-mpTiO_2_-PS_248_ synthesized using PEO-*b*-PS templates with higher molecular weights have a large pore size of 12.0 and 20.8 nm, respectively, and they also show much better activity compared to Pt-npTiO_2_ (Figure S8). The CO conversion of the WGS reaction over the three mesoporous catalysts at 250 °C follows the sequence Pt-mpTiO_2_-PS_173_ > Pt-mpTiO_2_-PS_120_ > Pt-mpTiO_2_-PS_248_, consistent with the order of pre-exponential factor (*A*) obtained from the kinetic studies (Table S5). Notably, the extent of self-enhancing activity decreases slightly with the increasing pore size of the mpTiO_2_ supports possibly due to the weakened confinement effect of the pore wall (Figure S9). Particularly, the activity of Pt-mpTiO_2_-PS_248_ with larger Pt NPs (2.01 ± 0.34 nm) decreases slightly during the cycling test because larger Pt particles can lead to a stronger MSI and further encapsulation of Pt NPs during the WGS reaction (Figure S4).

To gain insight into the unexpected cyclic performance and excellent stability of the Pt-mpTiO_2_ catalysts, XPS measurements were performed on the fresh and used Pt-mpTiO_2_-PS_120_ catalysts (Figure 3A and Table S3). The Ti 2p spectra show that the relative content of Ti^3+^ (∼458.3 eV) increases dramatically from 0.48 for the fresh Pt-mpTiO_2_-PS_120_ to 0.64 for the cyclic used catalysts, indicating the reduction of mpTiO_2_ support during the WGS reaction.^36,62^ The O 1s spectra show that the concentration of surface adsorbed oxygen species at oxygen vacancy (O_v_; ∼529.4 eV) for the used catalysts increases along with the dramatic down-shift of its binding energy, implying an increased concentration of positively charged O_v_ in the used Pt-mpTiO_2_-PS_120_.^36,63^ In addition, the electron paramagnetic resonance (EPR) measurements were also conducted to identify the concentration of O_v_ (Figure S13). In comparison with the fresh Pt-mpTiO_2_-PS_120_, the signal at a *g* value of 2.003 that is ascribed to the surface Ti^3+^ defect and single electron O_2_^–^ radical trapped O_v_ for the used catalyst becomes stronger (Figure S13a,b),^64^ further confirming the obvious increase of O_v_ concentration and agreeing well with the above-mentioned XPS results. In the XPS spectra for Pt 4f,^65,66^ the bands assigned to Pt^0^ (∼70.7 and 74.1 eV) for the used Pt-mpTiO_2_-PS_120_ shift to higher binding energy (Figure 3A), indicating the formation of positively charged Pt (Pt^δ+^, 0 < δ < 2) NCs and generation of numerous Ti^3+^-O_v_-Pt^δ+^ sites at the Pt/mpTiO_2_ interfaces. The formation of Ti^3+^-O_v_-Pt^δ+^ sites is due to the charge transfer from ultrasmall Pt NCs to mpTiO_2_, which is a typical electronic perturbation phenomenon in EMSI. However, the above findings and phenomena were not observed for Pt-npTiO_2_ (Figures S13c,d and S14 and Table S3), implying the negligible change of O_v_ concentration in the used Pt-npTiO_2_ probably due to larger Pt particles (∼3.38 nm). The increase of O_v_ concentration in the used Pt-mpTiO_2_-PS_120_ is beneficial to the dispersion of metal particles over the reducible TiO_2_ matrix^67^ and the dissociation of H_2_O molecules in the WGS reaction.

Considering that O_v_ is usually formed after the removal of lattice oxygen from crystalline metal oxides, the increase of O_v_ concentration of mpTiO_2_ in this study is probably due to the reduction of *in situ* generated H_2_ during the WGS reaction. To unravel the reason for the increase of O_v_ concentration in the used Pt-mpTiO_2_-PS_120_, H_2_ temperature-programmed desorption (H_2_-TPD) and H_2_ temperature-programmed reduction (H_2_-TPR) measurements were carried out on different catalysts. In comparison with the fresh Pt-npTiO_2_, the H_2_-TPD profile (Figure 3B) of the fresh Pt-mpTiO_2_-PS_120_ clearly shows a desorption peak for the spillover hydrogen at 607 °C, proving the obvious hydrogen spillover effect, which can greatly contribute to the increase of O_v_ concentration in mpTiO_2_. Moreover, a remarkable reduction peak was observed at 323 °C for mpTiO_2_ support in close interaction with Pt in the H_2_-TPR profile of Pt-mpTiO_2_-PS_120_ (Figure 3C), which indicates the easy reduction of adjacent Ti^4+^ to Ti^3+^ by the spillover hydrogen.^68,69^ Density functional theory (DFT) calculations (Figure 3D and Figure S15) further verify the superior transfer ability of dissociated hydrogen from small Pt NCs to TiO_2_, and lattice oxygen in the vicinity of Pt NCs confined in mpTiO_2_ can be more easily removed with a lower energy barrier of 0.2 eV. Therefore, it is clear that, during the WGS reaction, the strong reducing ability of spillover hydrogen generated at small Pt NCs is the main reason for the increase of O_v_ concentration in Pt-mpTiO_2_-PS_120_. According to the further comparison of the H_2_-TPD profiles for the used and fresh Pt-mpTiO_2_-PS_120_ (Figure 3B), it can be found that the spillover-hydrogen desorption peak at 607 °C for the used Pt-mpTiO_2_-PS_120_ decreases remarkably with the increase of the desorption peak for the chemisorbed hydrogen at 200–300 °C, indicating that the increase of O_v_ concentration in the used Pt-mpTiO_2_-PS_120_ can impede the hydrogen spillover and in turn accelerate the H_2_ desorption from the Pt NCs. Such an interesting and unusual tandem process is extremely favorable for an efficient WGS reaction.

Since steam is involved in the WGS reaction, the effect of steam on a Pt-mpTiO_2_-PS_120_ catalyst was also studied. The EPR measurement result (Figure S13a,e) shows that the steam treatment hardly increases the O_v_ concentration in Pt-mpTiO_2_-PS_120_. Interestingly, in the O 1s XPS spectra of the steam-treated Pt-mpTiO_2_-PS_120_, the peak ascribed to lattice oxygen (∼530.1 eV) shifts to high binding energy, implying that the lattice oxygen is positively charged by bonding with a hydrogen radical (H*) derived from H_2_O dissociation on the adjacent O_v_ (Figure S16). *In situ* diffuse reflectance Fourier transform infrared spectroscopy (DRIFTS) spectra of CO adsorption at 25 °C for the Pt-mpTiO_2_-PS_120_ (Figure S17) show that the Ti–O band (710 cm^–1^) displays a red-shift (696 cm^–1^) and becomes weaker with steam pretreatment, indicating that the Ti–O bond is more unstable after steam pretreatment. These results obviously confirm that the H* can greatly activate the lattice oxygen of mpTiO_2_ to generate active oxygen species that can be easily reduced to O_v_ by spillover hydrogen during the WGS reaction, agreeing well with H_2_-TPR, H_2_-TPD, and DFT calculation results. The lower desorption temperature of spillover hydrogen (460 °C) for the steam-treated Pt-mpTiO_2_-PS_120_ in contrast with that (607 °C) for the fresh Pt-mpTiO_2_-PS_120_ (Figure 3B) indicates the timely desorption of H_2_ due to the existence of hydroxyl derived from H_2_O dissociation,^70^ thus resulting in superior catalytic activity (Figure S16). According to the results mentioned above, it can be concluded that during the WGS reaction over Pt-mpTiO_2_-PS_120_ (Figure 3E), H* derived from H_2_O dissociation at the O_v_ sites can activate the adjacent lattice oxygen at the Pt/mTiO_2_ interfaces and promote the increase of O_v_ concentration by cooperating with the strong reducing ability of spillover hydrogen, and the newly generated O_v_ nearby the Pt/mTiO_2_ interfaces is more active.^71^ The newly generated O_v_ would successively promote the dissociation of H_2_O molecules and stabilization of Pt NCs, further facilitating the desorption of generated hydrogen and accelerating the WGS reaction process.

**Figure 4:**
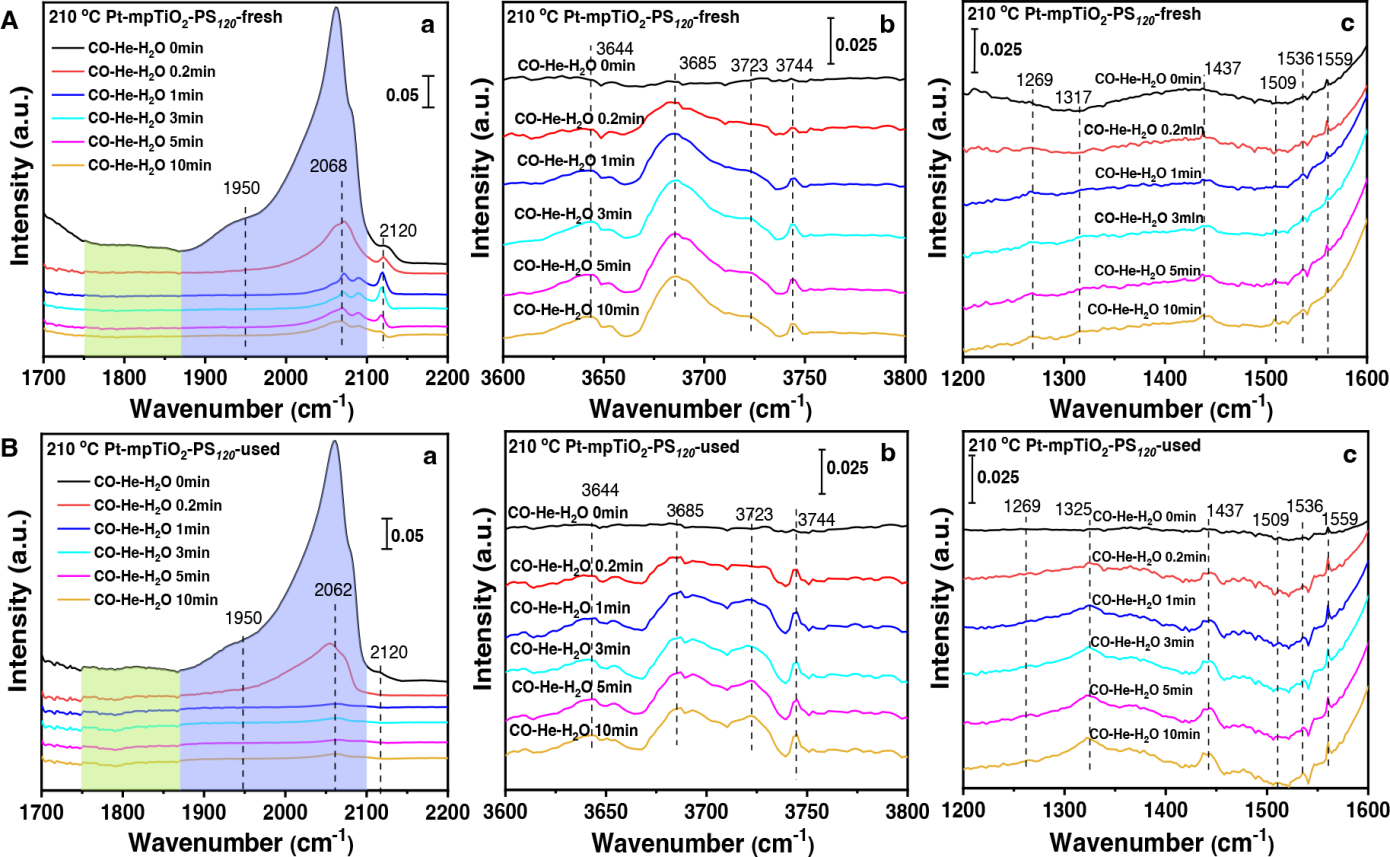
Catalytic mechanism of Pt-mpTiO_2_-PS_120_ toward the water–gas shift reaction. *In situ* time-resolved DRIFTS spectra of CO chemisorption on (A) fresh Pt-mpTiO_2_-PS_120_ and (B) used Pt-mpTiO_2_-PS_120_ after cyclic activity evaluation five times recorded in (a) 1700–2200 cm^–1^, (b) 3600–3800 cm^–1^, and (c) 1200–1600 cm^–1^ upon exposure to a H_2_O atmosphere at 210 °C as a function of reaction time (denoted as CO–He–H_2_O *x* min, *x* refers to the time of H_2_O injection).

To further study the catalytic mechanism of the Pt-mpTiO_2_-PS_120_ catalyst and clarify the superior catalytic performance of the used catalyst during the WGS reaction, *in situ* time-resolved carbon monoxide diffuse reflectance infrared Fourier transform spectroscopy (CO–DRIFTS) measurements were performed. According to *in situ* time-resolved DRIFTS spectra of CO chemisorption on Pt-mpTiO_2_-PS_120_ at 180 and 210 °C (Figure 4A and Figures S18–S24), it can be concluded that the WGS reaction over the Pt-mpTiO_2_-PS_120_ catalyst exclusively underwent a redox pathway rather than an associative pathway over the Ti^3+^-O_v_-Pt active sites. Interestingly, compared to the fresh Pt-mpTiO_2_-PS_120_, the band intensity corresponding to linearly adsorbed CO on Pt^δ−^ (1950 cm^–1^; Figure 4Ba) decreases along with the increase of the linearly adsorbed CO on Pt^0^ (2060 cm^–1^) after the CO adsorption on used Pt-mpTiO_2_-PS_120_ was flushed with He for 10 min, indicating the change of charge environment around Pt NCs. This result is in good agreement with the appearance of more Pt^δ+^ species shown in the XPS spectra (Figure 3A) and indicates the formation of the Ti^3+^-O_v_-Pt^δ+^ active sites in the used Pt-mpTiO_2_-PS_120_. The characteristic bands of adsorbed CO disappear more rapidly with the introduction of H_2_O at 210 °C (Figure 4Ba), suggesting the timely transfer of activated CO from used Pt-mpTiO_2_-PS_120_ due to the relatively weak CO chemisorption on the Pt^δ+^ NCs caused by the decreased back-donation of Pt d electrons into the 2π* antibonding orbital of CO.^72^ Moreover, the characteristic bands for Ti^3+^–OH increase obviously for the used catalyst (Figure 4Bb), indicative of the facilitated dissociation of H_2_O owing to the significant increase of O_v_ concentration. In addition, the intensities of HCO_3_^–^ (1437 cm^–1^) and CO_3_^2–^ bands (1325 cm^–1^) become stronger accompanied by the decrease of CO_2_^–^ bands (1269 and 1509 cm^–1^; Figure 4Bc). It demonstrates the facilitated redox pathway with faster generation of active carbonate intermediates over the abundant and efficient Ti^3+^-O_v_-Pt^δ+^ sites, accounting for the superior catalytic ability of the used Pt-mpTiO_2_-PS_120_ catalysts.

For comparison, *in situ* time-resolved DRIFTS spectra of CO chemisorption over Pt-npTiO_2_ were collected (Figures S18–S21 and Figures S25–S28), which disclosed the carboxyl associative pathway of the WGS reaction over Pt-npTiO_2_. The weakened bands for adsorbed CO and intermediates after introduction of H_2_O over the used catalyst (Figures S25–S28) reveal their decreased ability for CO activation and H_2_O dissociation due to the unfavorable overencapsulation of large Pt NPs induced by the SMSI, and this is further confirmed by the EELS analysis, which shows obvious signals of Ti *L*-edge and O *K*-edge at spots 2, 3, 4, and 5, similar to those at spot 1 (Figure S29). Therefore, a decreasing cyclic activity and stability toward the WGS reaction was observed, although there is no significant change in the size of Pt NPs (Figure S3).

**Figure 5:**
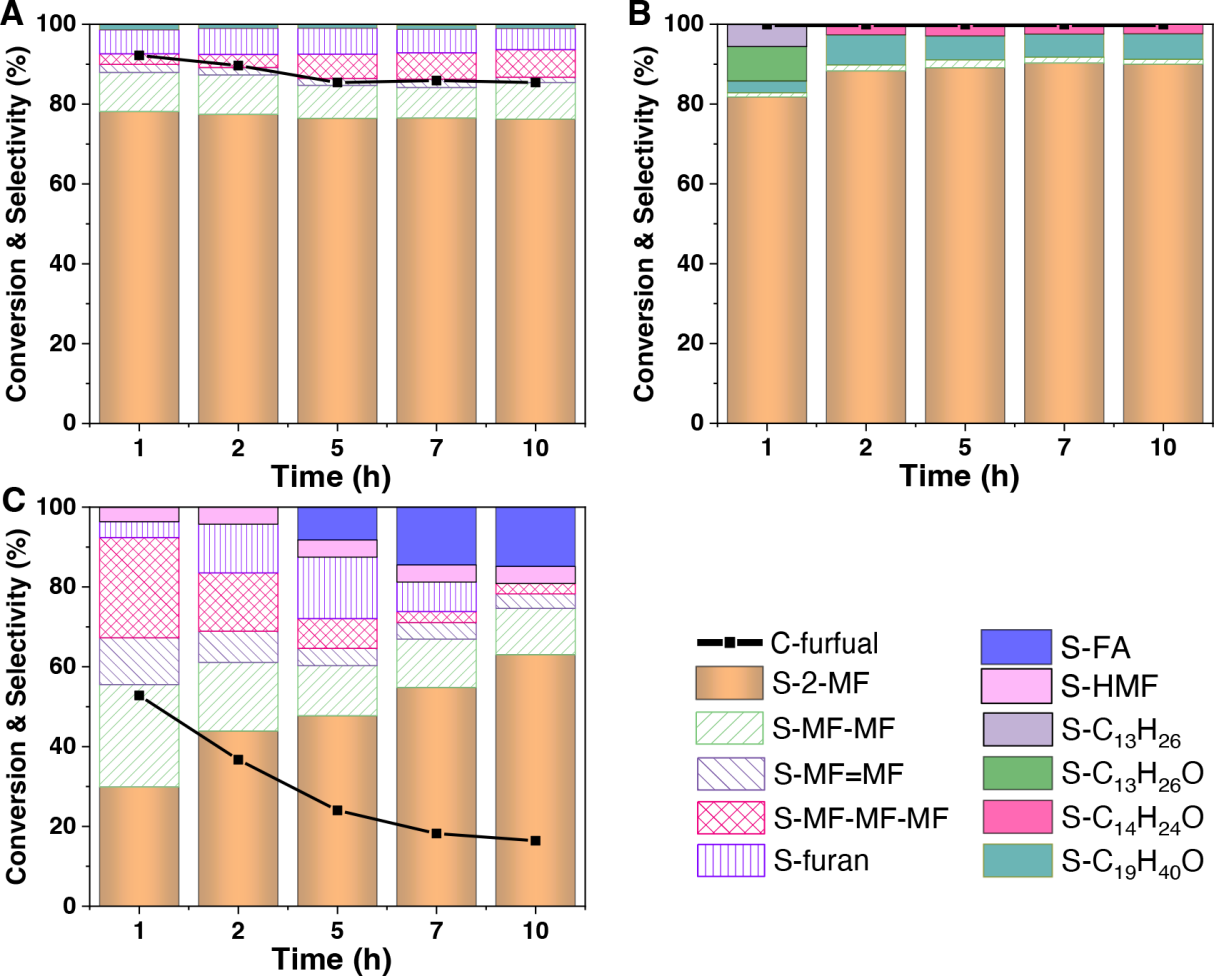
Catalytic activity of different catalysts toward hydrogenation of furfural to 2-methylfuran (MF). Furfural hydrogenation activity of (A) fresh Pt-mpTiO_2_-PS_120_, (B) used Pt-mpTiO_2_-PS_120_ after cyclic activity evaluation five times, and (C) fresh Pt-npTiO_2_ catalysts. C-furfural refers to the conversion of furfural during the hydrogenation reaction process. S-*x* is the selectivity of the product. and *x* refers to the name of the product.

The unique interfacial structure and catalytic behavior of Pt-mpTiO_2_-PS_*x*_ catalysts encourage us to extend their application in other heterogeneous catalysis reactions. Furfural is an indispensable intermediate for the sustainable preparation of high value-added platform molecules from biomass (cellulose). The effective transformation and removal of its C–O bond affects directly the purity and selectivity of the synthesized monomers, which is a key step for biomass upgrading and utilization, and the selective hydrogenation of furfural is strongly dominated by the distribution of active sites.^73−75^ The Pt-mpTiO_2_-PS_120_ displays better catalytic ability and stability than the Pt-npTiO_2_ catalyst toward the hydrogenation of furfural to 2-methylfuran (2-MF; Figure 5 and Table S6). During the continuous activity evaluation process, stable catalytic activity with high conversion (∼90%) of furfural and high selectivity (∼80%) toward the target product 2-MF were observed for the fresh Pt-mpTiO_2_-PS_120_ catalyst. The result is consistent with the findings in the previous report;^76^ namely, Pt nanoclusters (NCs) can selectively activate the C–O bond scission, whereas metallic Pt NPs can promote both C–O activation and ring hydrogenation, thus resulting in a lower selectivity to 2-MF. In addition, the activity of used Pt-mpTiO_2_-PS_120_ is further improved with the stability maintained, and the furfural conversion and selectivity toward 2-MF reached as high as ∼100% and ∼90%, respectively, which are superior to most supported metal catalysts reported in the previous work under similar test conditions, even bimetallic catalysts (Table S7). The decreased electron density of these Pt NCs due to the EMSI promotes the furfural adsorption and contributes to the improved activity. Moreover, the used Pt-mpTiO_2_-PS_120_ after the WGS reaction also possesses superior catalytic ability in photocatalytic degradation of organic pollutants (Figure S30) with almost twice the degradation rate compared to the fresh one.

## Conclusions

In summary, a highly stable supported metal cluster (SMC) catalyst with rich active Pt/mpTiO_2_ interfaces was designed by confinement of Pt NCs in the uniform mesopores of mesoporous titania, and an unusual self-enhancing activity was discovered for this novel SMC catalyst. The self-enhancing activity of the Pt-mpTiO_2_ was found to stem from the *in situ* generated Ti^3+^-O_v_-Pt^δ+^ active sites that play important roles in facilitating the dissociation of H_2_O, the transfer of activated CO, and the desorption of generated hydrogen and eventually significantly accelerate the WGS reaction via the redox pathway. It is the collective effects of the strong reducing ability of spillover hydrogen over Pt NCs, the easy activation of lattice oxygen on mpTiO_2_, and the electronic metal–support interaction that allow for the formation of Ti^3+^-O_v_-Pt^δ+^ active sites at the metal/metal oxide interface. The confinement effect of mpTiO_2_ support contributes to the stable dispersion of ultrafine Pt NCs and prevents their migration and growth, resulting in an outstanding catalytic stability in the WGS reaction with activity of up to 13.5 times higher than reported similar catalysts. The Pt-mpTiO_2_ catalysts also show superior performance toward the selective hydrogenation of furfural to 2-methylfuran compared to Pt-npTiO_2_. The discovery of the self-enhancing activity and the findings about the underlying mechanism of mpTiO_2_ supported Pt NCs can serve as a useful guideline in exploring a variety of supported metal nanoclusters with improved interfacial activity in different fields, including catalysis, sensing, energy conversion, and storage.

## Experimental Section

### Chemicals and Materials

All of the chemicals were analytical grade. Monomethyl poly(ethylene oxide) (*M*_w_: 5000 g·mol^–1^), chloroplatinic acid hexahydrate (H_2_PtCl_6_·6H_2_O), furfural, Rhodamine B, and the commerical TiO_2_ (anatase phase) were purchased from Aladdin Chemical Reagent Co. Ltd. Tetrabutyl orthotitanate (TBOT), tetrahydrofuran (THF), and ethanol (EtOH) were purchased from Sino-Pharm Chemical Reagent Co. Ltd. Deionized water was used in the whole experimental process.

### Synthesis of Mesoporous TiO_2_ Support

The amphiphilic poly(ethyl oxide)-*block*-polystyrene diblock copolymers with different hydrophobic chain length (PEO-*b*-PS_*x*_) templates prepared by the atom transfer radical polymerization (ATRP) method were used for the synthesis of mesoporous TiO_2_ (mpTiO_2_) via the solvent evaporation induced coassembly (EICA) approach (Supplementary Scheme 1, steps 1–4). In a typical synthesis process, 100 mg of PEO-*b*-PS was first dissolved in 5 mL of THF to form a homogeneous solution, and 150 μL of concentrated hydrochloric acid (35%–37% HCl) and 150 μL of concentrated acetic acid were added dropwise into the above solution under magnetic stirring. After stirring for 5 min, 400 μL of TBOT was added dropwise into the resultant solution. After stirring for another 2.0 h, the solution was poured into Petri dishes to evaporate solvent at 25 °C for 24 h, followed by sequential heating at 40 °C for 24 h to remove the solvent completely and at 100 °C for another 24 h to fix the structure. The transparent film was scraped and crushed into faint yellow powders which were first calcined in a tube furnace under a N_2_ atmosphere at 350 °C for 3 h with a heating rate of 1 °C/min and then in the air at 450 °C for 30 min with a heating rate of 5 °C/min. By using the PEO-*b*-PS template with different PS lengths (PEO-*b*-PS_120_ with Mn = 17484 g/mol and polydispersity index (PDI) = 1.13, PEO-*b*-PS_173_ with Mn = 23038 g/mol and PDI = 1.11, and PEO-*b*-PS_248_ with Mn = 30797 g/mol and PDI = 1.12), crystalline ordered mesoporous TiO_2_ with different pore sizes can be obtained and denoted as mpTiO_2_-PS_*x*_, wherein *x* refers to the polymerization degree of the PS segment. For comparison, nonporous TiO_2_ (designed as npTiO_2_) was synthesized according to the same procedure but without adding the template.

### Synthesis of Pt-mpTiO_2_ Catalyst

Pt-mpTiO_2_ catalysts were prepared by the wet-impregnation method (Supplementary Scheme 1 step 5). Typically, 0.1 g of mpTiO_2_-PS_*x*_ was dispersed into 5 mL of H_2_O by sonication. Then, 1 mL of H_2_PtCl_6_·6H_2_O aqueous solution (0.01 mM; corresponding to a 2 wt % loading amount of Pt) was added to the mixture, followed by stirring at 25 °C for 12 h. Finally, the mixture was centrifuged, and the product was washed with H_2_O two times followed by EtOH washing two times and vacuum drying at 50 °C for 8 h. For comparison, the Pt-npTiO_2_ catalyst was also prepared via the above procedure. Subsequently, the obtained samples were treated in a H_2_/Ar atmosphere (1:19, v/v) at 300 °C for 2 h with a ramp of 5 °C/min, followed by cooling to room temperature under a N_2_ atmosphere. The obtained catalysts were labeled as Pt-mpTiO_2_-PS_*x*_ and Pt-npTiO_2_, respectively.

### Characterization and Measurement

The powder X-ray diffraction (XRD) measurements were conducted using a Bruker D4 X-ray diffractometer (Germany) with Ni-filtered Cu Kα radiation (40 kV, 40 mA), and small-angle X-ray scattering (SAXS) patterns were collected using a Nanostar U SAXS system (Bruker, Germany). Thermogravimetric analysis (TGA) was conducted via a TA Instruments SDT Q600 analyzer (America) from 25 to 600 °C with a ramp rate of 10 °C/min. Field-emission scanning electron microscopy (FESEM) was performed on the Zeiss Ultra 55 FESEM (Germany). Transition electron microscopy (TEM) and high angle annular dark-field scanning transmission electron microscopy (HAADF-STEM) were carried out on a Tecnai G2 F20 S-Twin microscope (FEI, America). Size statistics of the Pt NCs or Pt NPs in the catalysts were made using ImageJ software across more than 100 points on the HAADF-STEM image, and the mean value and standard deviation were calculated for comparison. Aberration-corrected high angle annular dark-field scanning transmission electron microscopy (ac-HAADF-STEM) and electron energy-loss spectroscopy (EELS) were carried out on a FEI-Titan Cubed Themis G2 300 (The Netherlands). N_2_ adsorption–desorption measurements were carried out at 77 K on a Micromeritics Tristar 2420 analyzer. X-ray photoelectron spectroscopy (XPS) measurements were performed on an AXIS Ultra DLD X-ray photoelectron spectrometer with a MONO Al source (Shimadzu Corp). The electron paramagnetic resonance (EPR) spectra were conducted at room temperature on a Bruker EMX-10/12 spectrometer with 9.5 GHz X-band. H_2_ temperature-programmed desorption (H_2_-TPD) was conducted on a Micromeritics AutoChem II 2920 with an online mass spectrometer (MS). H_2_ temperature-programmed reduction (H_2_-TPR) was conducted on a Micromeritics AutoChem II 2920 equipped with a thermal conductivity detector (TCD). Loading amounts of Pt in the catalysts were detected on a Thermo Scientific iCAP 7400 inductively coupled plasma atomic emission spectrometer (ICP-AES).

### Catalytic Tests for Water–Gas Shift (WGS) Reaction

The WGS reaction was performed on a continuous flow fixed-bed reactor from 120 to 300 °C under atmospheric pressure. During each test, 50 mg of catalyst was placed into the U-type quartz tube with an interior diameter of 6 mm. Water was injected into the heated gas feed line (160 °C) using a calibrated syringe pump, and the generated steam was mixed with the CO/N_2_ gas stream before entering the reactor. The reactant gas consisted of 2% CO, 8% H_2_O, and 90% N_2_ with total flow rate of 50 mL/min, and the weight hourly space velocity (WHSV) was maintained at 60 000 mL g_cat_^–1^ h^–1^. The outlet gas was analyzed online using a gas chromatograph equipped with a thermal conductivity detector (TCD) and a fire ionization detector (FID) after the condensation of water at the exit of the reactor. During each test, the catalyst was pretreated in a N_2_ atmosphere at 300 °C for 30 min and then cooled down to room temperature. At each reaction temperature (120, 150, 200, 250, and 300 °C), the test was maintained for 30 min. The cyclic catalytic activity evaluations of Pt-mpTiO_2_ and Pt-npTiO_2_ were conducted continuously five times under the same heating procedures. Catalytic stability evaluation was carried out at 250 °C for a total of 96 h with a period of 12 h. During the study, no methane was detected, and the conversion of CO was calculated using the following equation:
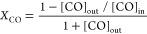


For the calculation of mass specific activity (*k*) and metal normalized activity (*R*), the total CO conversion was kept below 10%. The mass specific activity and metal normalized activity were calculated using the following equations:
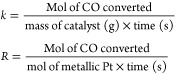


### Steam Treatment of Pt-mpTiO_2_-PS_120_

In order to identify the impact of H_2_O on the enhanced O_v_ correlating with the excellent cyclic performance of the used mesoporous catalyst, Pt-mpTiO_2_-PS_120_ was treated in a H_2_O/N_2_ (8%/92%, v/v) atmosphere with a total flow rate of 50 mL/min for 30 h and was labeled as the Pt-mpTiO_2_-PS_120_-H_2_O pretreatment.

### DRIFTS Experiments

*In situ* diffuse reflectance Fourier transform infrared spectroscopy (DRIFTS) was conducted using an *in situ* diffuse-reflectance cell on the Nicolet 6700 (ThermoFisher) equipped with an MCT detector. The detailed process is shown as follows.

*In situ* time-resolved DRIFTS spectrum analysis was performed to identify the catalytic performance of the interfacial active sites. About 50 mg of catalyst sample was filled into the reactor, pretreated in a He atmosphere at 300 °C for 30 min. Then, the sample was slowly cooled to 180 and 210 °C, respectively, followed by the chemisorption of CO in a 1% CO/He atmosphere for 10 min and subsequent flushing with He for 10 min. Finally, H_2_O was injected into the reactor, and the time-resolved DRIFTS spectra were recorded using a 64-scan quick sweep mode with a resolution of 4 cm^–1^.

*In situ* DRIFTS spectrum analysis was also performed to reveal the influence of H_2_O on promoting the activity of the mesoporous catalyst. The catalyst sample was placed into the reactor, pretreated in a He atmosphere at 300 °C for 1 h or in a H_2_O/He atmosphere at 300 °C for 30 min, followed by flushing with He for 30 min. After cooling down to 25 °C, 1% CO/He was introduced for 10 min. Then, the DRIFTS spectra were collected after flushing with He for 10 min.

### DFT Calculations

We have employed first principles^77,78^ to perform all spin-polarization density functional theory (DFT) calculations within the generalized gradient approximation (GGA) using the Perdew–Burke–Ernzerhof (PBE)^79^ formulation. We have chosen the projected augmented wave (PAW) potentials^80,81^ to describe the ionic cores and take valence electrons into account using a plane wave basis set with a kinetic energy cutoff of 450 eV. Partial occupancies of the Kohn–Sham orbitals were allowed using the Gaussian smearing method and a width of 0.05 eV. The electronic energy was considered self-consistent when the energy change was smaller than 10^–6^ eV. A geometry optimization was considered convergent when the energy change was smaller than 0.03 eV Å^–1^. The vacuum spacing in a direction perpendicular to the plane of the structure is 15 Å. The Brillouin zone integration is performed using 3 × 3 × 1 Monkhorst–Pack k-point sampling for a structure. The free energy was calculated using the equation
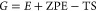
where *G*, *E*, ZPE, and TS are the free energy, total energy from DFT calculations, zero-point energy, and entropic contributions, respectively.

### Hydrogenation of Furfural

A tandem reactor coupled with GC-MS (Froniter Rx-3050-Agilent7890B-5977B) was used to evaluate the catalytic performance of nascent and used catalysts for furfural hydrogenation. Twenty-five milligrams of the catalyst was placed in the second reactor tube in a flat-temperature regime of 300 °C; 10 mL of H_2_ + 41.5 mL of He continuously passed through the catalyst layer (10 mm in height and 2 mm in diameter) during the whole test. Subsequently, 5 μL of furfural liquid (99% AR, Aladdin) was injected from the top of first reactor and evaporated at 300 °C immediately, and it took about 5 s for the furfural vapor to pass through the catalyst completely. The temperature program of the chromatographic column is as follows: initial hold at 40 °C for 5 min, then increase to 250 °C with a heating rate of 5 °C/min and hold at 250 °C for 10 min. The injection interval was 1 h, and the test times was equal to the total time of hydrogen exposure on the catalyst. The stability of the catalyst in a long-term hydrogen atmosphere was evaluated by the selectivity and activity of this transient probe reaction without consideration of catalyst poisoning and carbon deposition.

### Photocatalytic Degradation of Rhodamine B

The photocatalytic performance of the Pt-mpTiO_2_-PS_120_ catalyst was tested by the model reaction of photocatalytic degradation of Rhodamine B (RHB). A xenon lamp (300 W) coupled with a 420 nm filter was used as the light source. A total of 12.5 mg of the catalyst was added into 25 mL of RhB solution (10 mg/L), followed by continuous magnetic stirring to keep a homogeneous system. Before the experiment, the reactor was placed in the dark for 4 h to ensure the adsorption equilibrium of RhB on the catalyst materials. During the experiment, the absorbance of RhB solution at 552 nm was tested using a UV–vis spectrometer every 10 min. The degradation rate calculated by the following equations was used to describe the photocatalytic ability of Pt-mpTiO_2_-PS_120_:
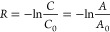
where *C*_0_ is the initial concentration of RhB, *C* is the concentration of RhB at time *t*, *A*_0_ is the initial absorbance of RhB solution, and *A* is the absorbance of RhB solution at time *t*.

## Supporting Information Available

Extra structural characterizations, including XRD patterns, SAXS patterns, N_2_ adsorption–desorption isotherms, additional FESEM images, supported evidence for synthesis, catalytic performance, and characterization of ordered mesopore confined Pt nanoclusters (PDF)
