# Supplementary material for: Ordered Mesopore Confined Pt Nanoclusters Enable Unusual Self-Enhancing Catalysis
Source: ACS Cent Sci. 2022 Dec 16;8(12):1633–45. doi: 10.1021/acscentsci.2c01290 (PMC9801509; doi:10.1021/acscentsci.2c01290)
Supplement: Supplementary file 1 — oc2c01290_si_001.pdf [file oc2c01290_si_001.pdf]

Supporting Information

Ordered Mesopore Confined Pt Nanoclusters Enable Unusual Self-Enhancing Catalysis

Meiqi Gao[a#], Zhirong Yang[b#], Haijiao Zhang[c], Junhao Ma[a], Yidong Zou[a]*, Xiaowei Cheng[a], Limin Wu[d]*, Dongyuan Zhao[a], Yonghui Deng[a]*

[a]Department of Chemistry, Department of Gastroenterology and Hepatology, Zhongshan Hospital, State Key Laboratory of Molecular Engineering of Polymers, Shanghai Key Laboratory of Molecular Catalysis and Innovative Materials, *i*ChEM, Fudan University, Shanghai 200433, China

[b]State Key Laboratory of Chemical Engineering, East China University of Science and Technology, Shanghai, 200237, China

[c]Institute of Nanochemistry and Nanobiology, School of Environmental and Chemical Engineering, Shanghai University, Shanghai 200444, P. R. China

[d]Institute of Energy and Materials Chemistry, Inner Mongolia University, Hohhot 010021, China

*E-mail: ydzou@fudan.edu.cn (Y. Zou); wlm@imu.edu.cn (L. Wu); yhdeng@fudan.edu.cn (Y. Deng)

[#]M. Gao and Z. Yang contributed equally to this work.

Supplementary figures and tables

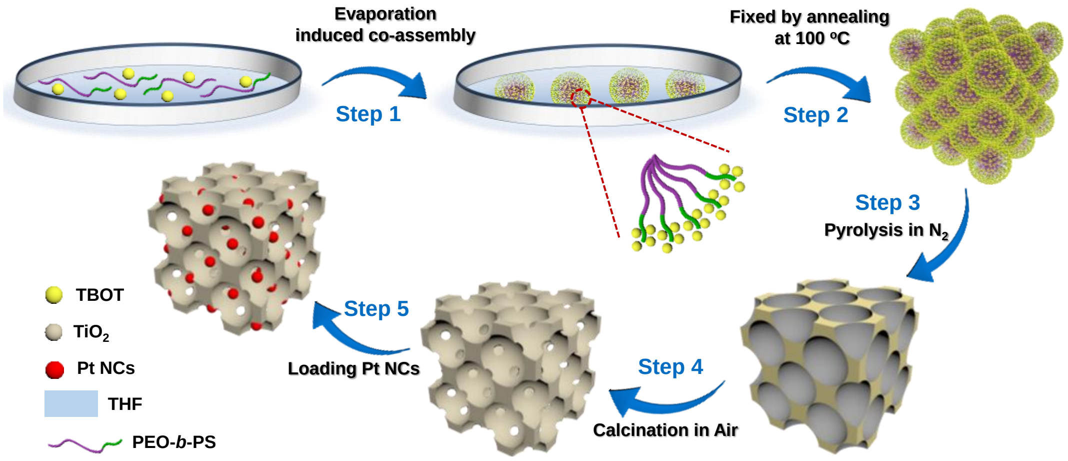

Scheme S1. The synthesis process of the mesoporous titania (mpTiO_2) and Pt-mpTiO_2. Step 1: The partially hydrolyzed titanium precursor (TBOT) co-assemble with PEO-*b*-PS copolymers into spherical micelles with the core of hydrophobic PS segments and the hybrid shell of PEO/titanium oxides as THF evaporates from the precursor solution; Step 2: The spherical composite micelles further organize into face-centered cubic mesostructures along with the further evaporation of THF, and the mesostructured organic/inorganic composites are then treated at 100 °C for solidification; Step 3: The carbon/TiO_2 composites with ordered mesostructure are formed after carbonization in N_2 atmosphere at 350 °C for 3 h; Step 4: The crystalline ordered mesoporous TiO_2 can be obtained after calcination in air atmosphere at 450 °C for 30 min to remove the supporting carbon; Step 5: the Pt-mpTiO_2 catalysts are prepared by the wet-impregnation method.

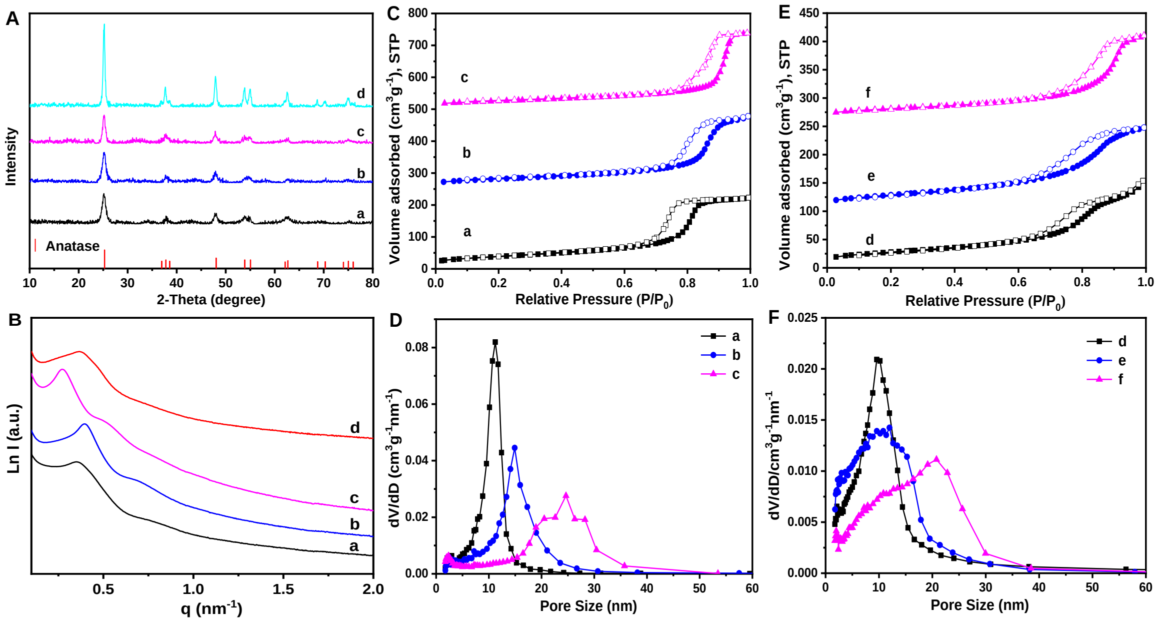

Figure S1. (**A**) XRD patterns of the (a) mpTiO$_2$-PS$_{120}$ obtained by using PEO-*b*-PS$_{120}$, (b) mpTiO$_2$-PS$_{173}$ obtained by using PEO-*b*-PS$_{173}$, (c) mpTiO$_2$-PS$_{248}$ obtained by using PEO-*b*-PS$_{248}$, and (d) npTiO$_2$; (**B**) SAXS patterns of the (a) mpTiO$_2$-PS$_{120}$, (b) mpTiO$_2$-PS$_{173}$, (c) mpTiO$_2$-PS$_{248}$, and (d) fresh Pt-mpTiO$_2$-PS$_{120}$; (**C, E**) N_2 adsorption-desorption isotherms and (**D, F**) pore size distribution curves of (a, b, c) crystalline mpTiO$_2$-PS$_x$ (a: mpTiO$_2$-PS$_{120}$, b: mpTiO$_2$-PS$_{173}$, c: mpTiO$_2$-PS$_{248}$) as well as (d, e, f) fresh Pt-mpTiO$_2$-PS$_x$ (d: Pt-mpTiO$_2$-PS$_{120}$, e: Pt-mpTiO$_2$-PS$_{173}$, f: Pt-mpTiO$_2$-PS$_{248}$).

The mesoporous TiO$_2$ (mpTiO$_2$-PS$_x$, *x=120*, *173*, or *248*) templated from poly(ethyl oxide)-block-polystyrene copolymers with different hydrophobic chain length (PEO-*b*-PS$_x$) display a series of similar XRD diffraction peaks assigned to anatase phase TiO$_2$ (Figure. S1Aa-c), which is the same as npTiO$_2$ (Figure S1Ad). The SAXS patterns (Figure S1Ba-c) reveal that all of the synthesized mpTiO$_2$ samples possess the ordered face-centered cubic (*fcc*) mesostructure with the space group of $Fm\bar{3}m$.[1,2] FESEM images (Figure S2A) of the mpTiO$_2$-PS$_{120}$ display an ordered mesoporous structure with uniform spherical mesopores, and such an ordered mesostructure with well-connected pores, uniform pore size and high specific surface area was further confirmed by the

corresponding N_2 adsorption-desorption measurement (Figure S1Ca, Da and Table S1). The SEM images (Figure S2B, C) and N_2 adsorption-desorption measurement results (Figure S1Cb,c, 1Db,c, and Table S1) of the mpTiO_2-PS_{173} and mpTiO_2-PS_{248} also indicate a highly-ordered mesostructure. The spotty diffraction rings in the selected area electron diffraction (SAED) patterns reveal a poly-crystalline framework of the mpTiO_2 (Figure S2D, insert), and the lattice spacing of 0.352 nm observed in the high-resolution TEM (HRTEM) image is assigned to the (101) plane of the typical anatase phase TiO_2 (Figure S2D). On contrary, no obvious porous structure can be observed on the synthesized npTiO_2 (Figure S3A).

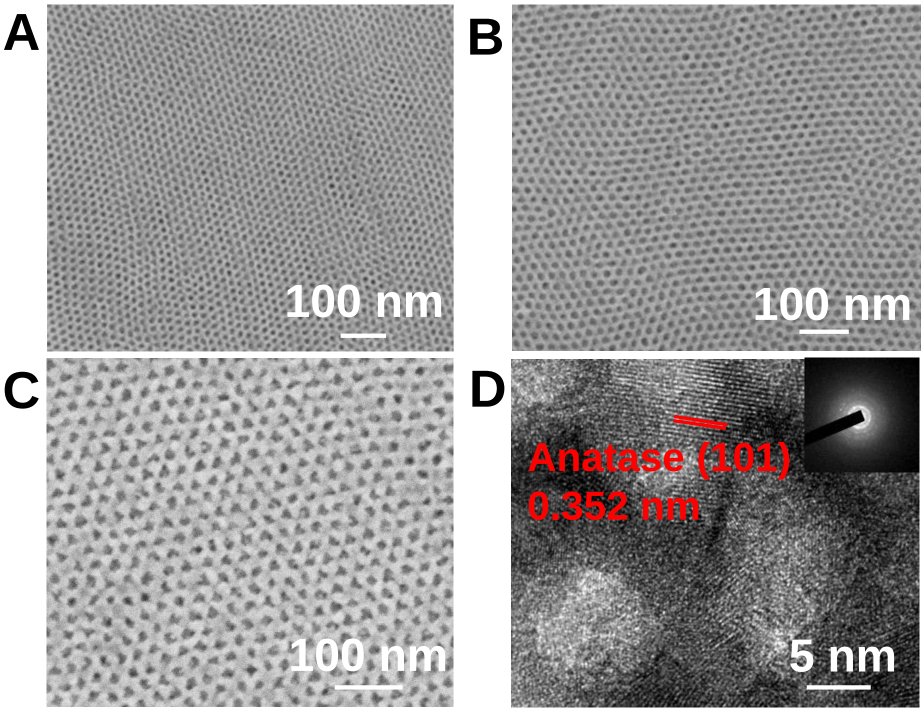

Figure S2. FESEM images of the (**A**) crystalline mpTiO_2-PS_{120}, (**B**) crystalline mpTiO_2-PS_{173}, and (**C**) crystalline mpTiO_2-PS_{248}; (**D**) HRTEM images of the crystalline mpTiO_2-PS_{248}.

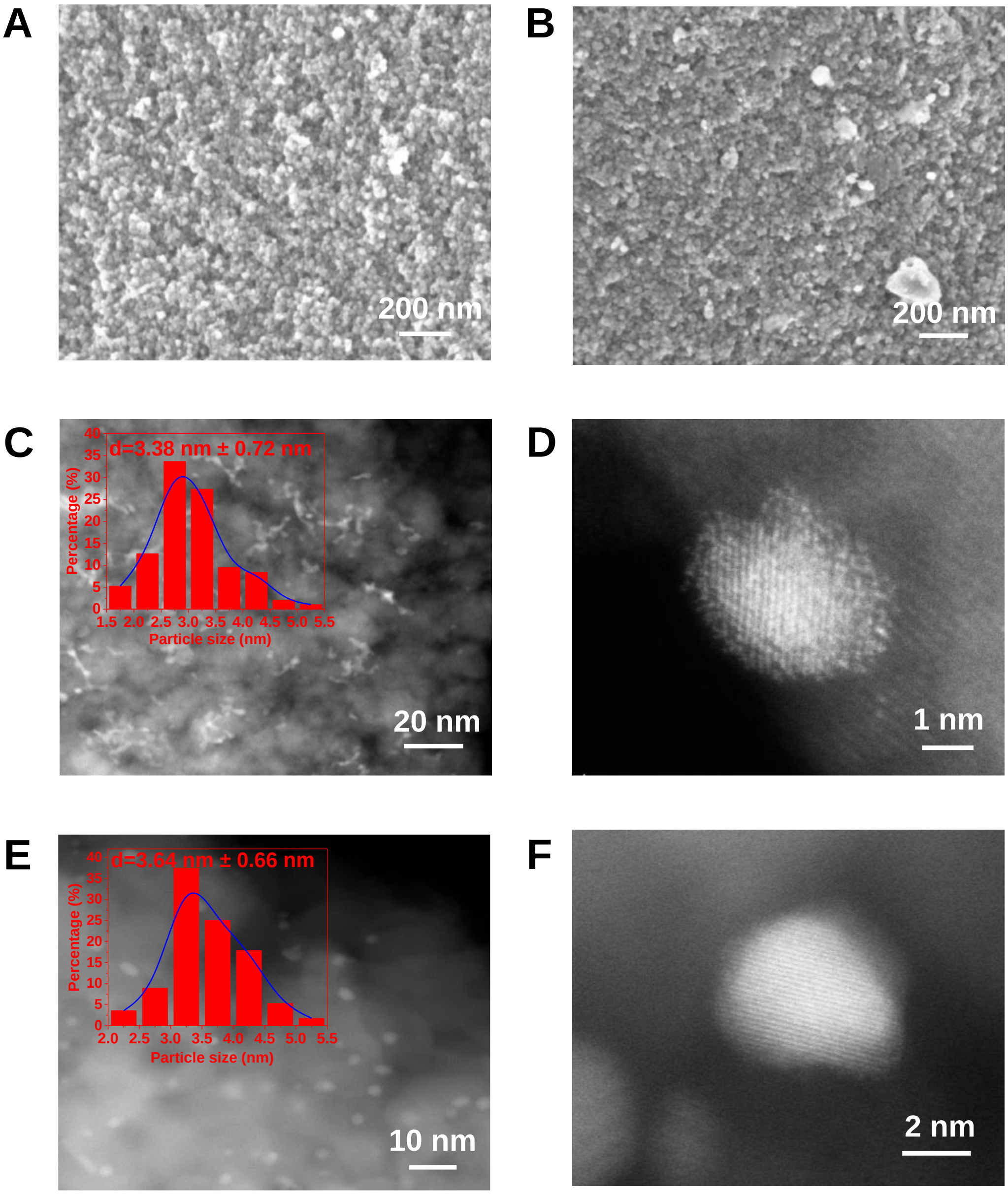

Figure S3. (**A**) FESEM image of the $npTiO_2$; (**B**) FESEM, (**C**) HAADF-STEM, and (**D**) Ac-HAADF-STEM images of the fresh Pt-$npTiO_2$; (**E**) HAADF-STEM and (**F**) Ac-HAADF-STEM images of the used Pt-$npTiO_2$ after cyclic activity evaluation five times.

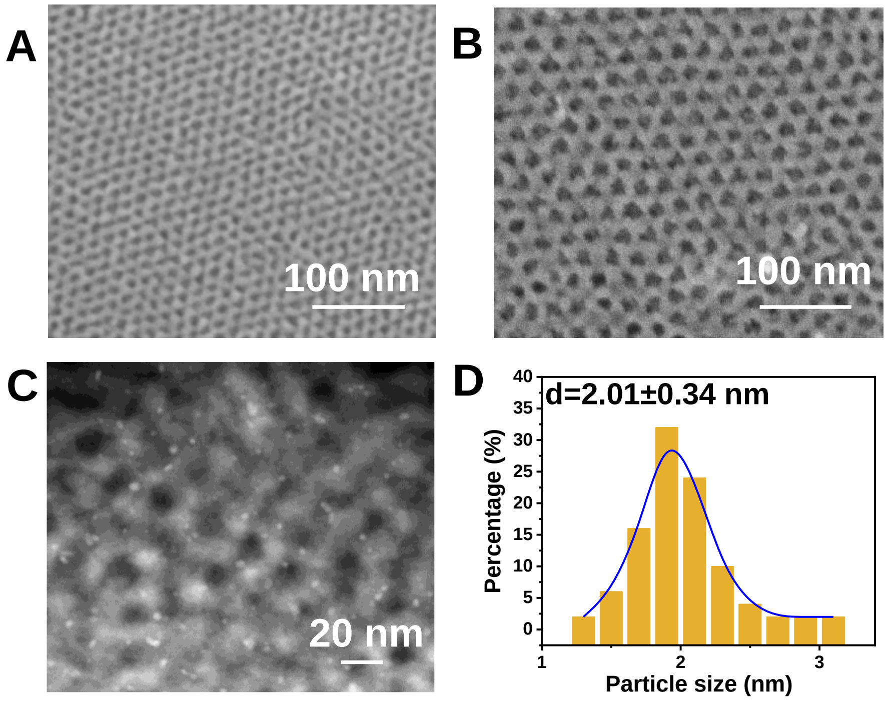

Figure S4. FESEM images of the (**A**) Pt-mpTiO_2-PS$_{173}$ and (**B**) Pt-mpTiO_2-PS$_{248}$; (**C**) HAADF-STEM images of the Pt-mpTiO_2-PS$_{248}$; (**D**) particle size distribution of Pt NPs on the Pt-mpTiO_2-PS$_{248}$.

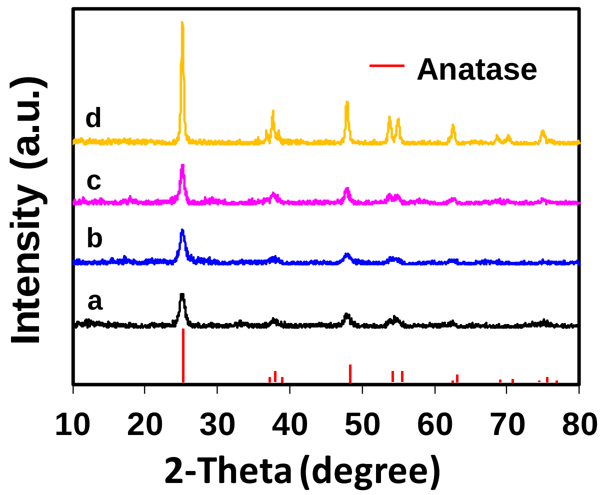

Figure S5. XRD patterns of the (a) Pt-mpTiO_2-PS$_{120}$, (b) Pt-mpTiO_2-PS$_{173}$, (c) Pt-mpTiO_2-PS$_{248}$, and (d) Pt-npTiO_2.

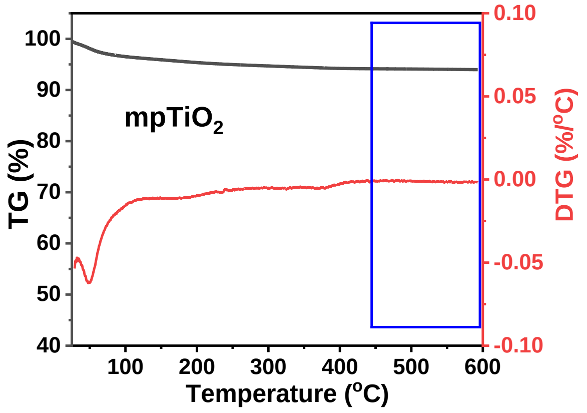

Figure S6. TGA curve of $mpTiO_2$ after calcination in air at 450 °C for 30 min

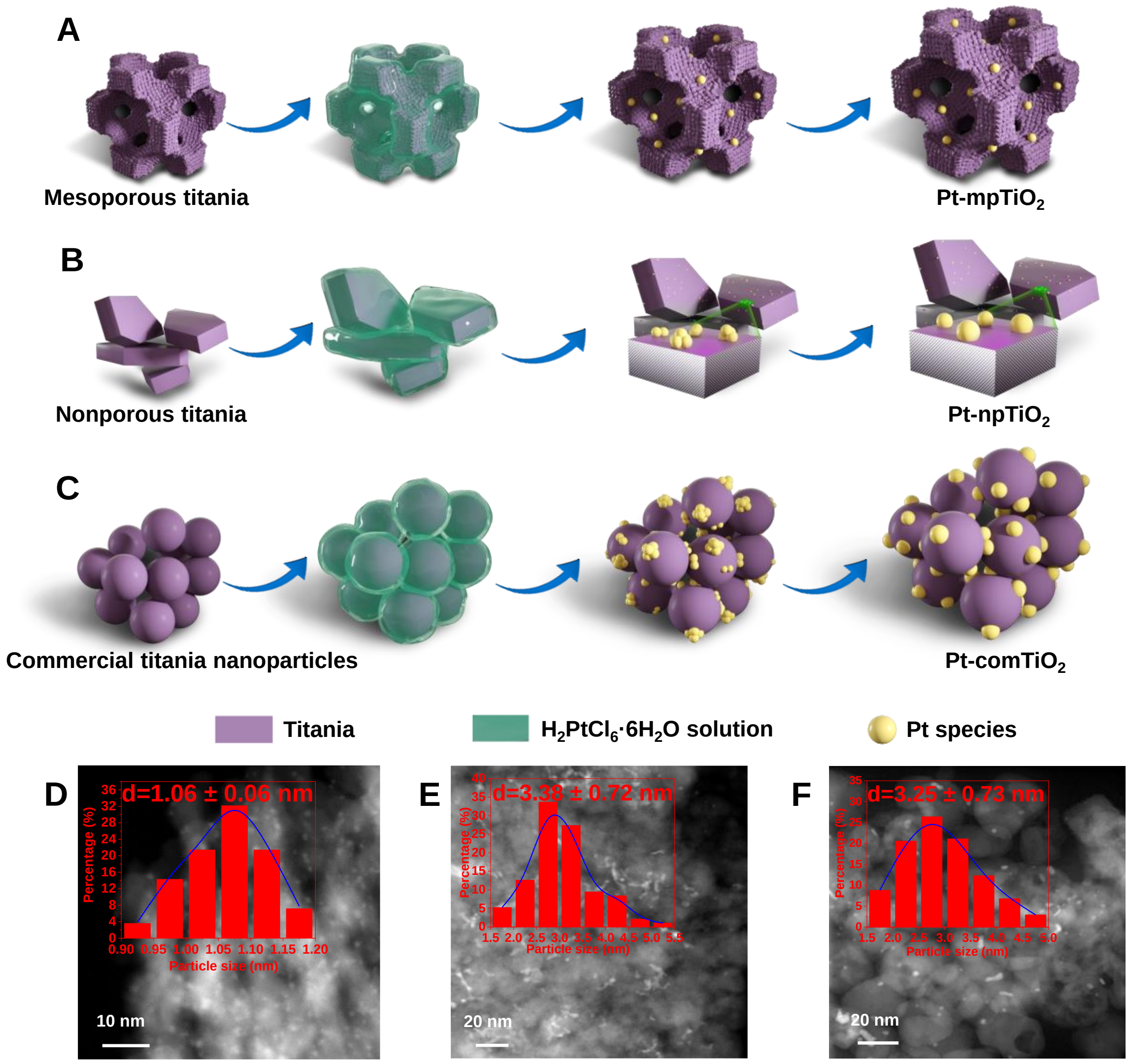

Figure S7. Evolution of Pt species on (A) $mpTiO_2$, (B) as-synthesized $npTiO_2$ and (C) $comTiO_2$ powders during wet-impregnation process. HAADF-STEM images of the obtained (D) Pt-$mpTiO_2$-PS_{120}, (E) Pt-$npTiO_2$ and (F) Pt-$comTiO_2$ catalysts.

Pt species were loaded on the $mpTiO_2$, as-synthesized $npTiO_2$ and $comTiO_2$ powders, respectively, by the wet-impregnation method using chloroplatinic acid hexahydrate ($H_2PtCl_6 \cdot 6H_2O$) as precuror. Figure. S6A-C schematically shows the evolution of Pt species on these different supports during respective synthesis processes. Since the rough surface is beneficial to the deposition and adsorption of guest molecules,[3] after vacuum drying, the precursor of Pt species dispersed quite evenly in the pore channels of $mpTiO_2$ due to the rough interior surface made of randomly packed and sintered TiO_2 nanoparticles.

Such a uniform dispersion of Pt species can still be maintained during H_2 reduction by right of the confinement effect of the rough wall of mpTiO_2, resulting in the homogeneous distribution of Pt NCs with ultrafine size of 1.06 ± 0.06 nm (Figure S7D). However, in the wet-impregnation method, the Pt precursor cannot be distributed uniformly on the as-synthesized npTiO_2 and comTiO_2 powders due to lack of confinement effect and, instead, they tend to migrate on the surface during the vacuum drying process and sintered into large nanoparticles of 3.38 ± 0.72 and 3.25 ± 0.73 nm (Figure S7E, F), respectively, during H_2 reduction process.

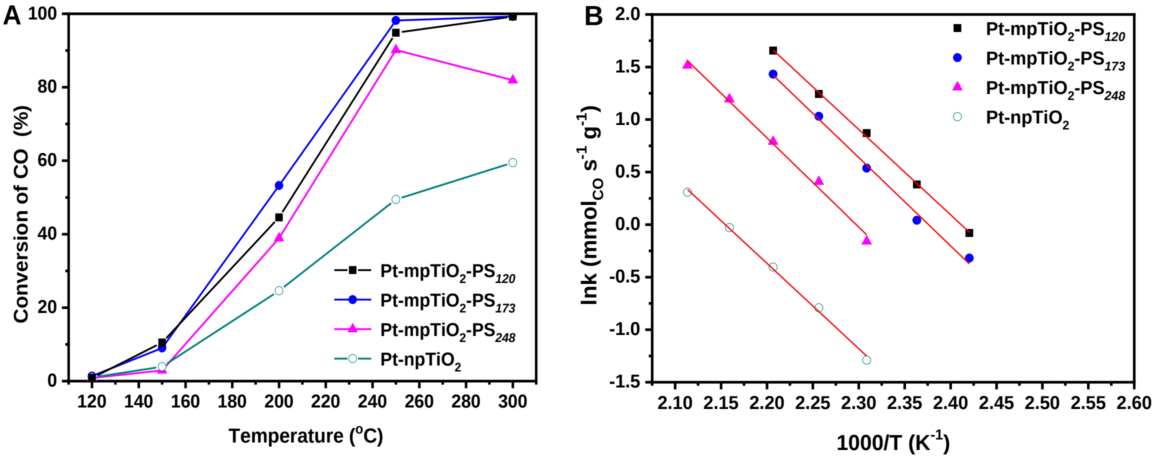

Figure S8. (**A**) CO conversion curves as a function of temperature and (**B**) Arrhenius plots of WGS reaction over different Pt-TiO_2 catalysts.

It can be seen from Figure S8A that all the mesoporous catalysts (Pt-mpTiO_2-PS_{120}, Pt-mpTiO_2-PS_{173} and Pt-mpTiO_2-PS_{248}) have the superior catalytic performance compared to the Pt-npTiO_2 catalyst. Among the three mesoporous catalysts, the Pt-mpTiO_2-PS_{173} possesses the best catalytic performance with a CO conversion of 98.2% at 250 °C; while the Pt-mpTiO_2-PS_{248} shows relatively poor activity with the CO conversion of 90.2% at 250 °C, demonstrating the significance of the mesoporous structure with suitable pore size during the WGS reaction.

According to the Arrhenius plots (Figure S8B), the apparent activation energies (*Ea*) and pre-exponential factors (*A*) of WGS reaction over different catalyst samples are listed in Table S5. It can be concluded that the activation energies of WGS reaction over different catalysts are very similar, while the pre-exponential factors give the following sequence: Pt-mpTiO_2-PS_{173} (4.7E8) > Pt-mpTiO_2-PS_{120} (3.2E8) > Pt-mpTiO_2-PS_{248} (3.0E8) > Pt-npTiO_2 (4.0E7), consistent with their activity. It indicates that the accelerated gas diffusion rate and high interaction efficiency of gas molecules with active sites by virtue of the interconnected pores and highly dispersed Pt species of the Pt-mpTiO_2-PS_x can greatly facilitate the WGS reaction activity.

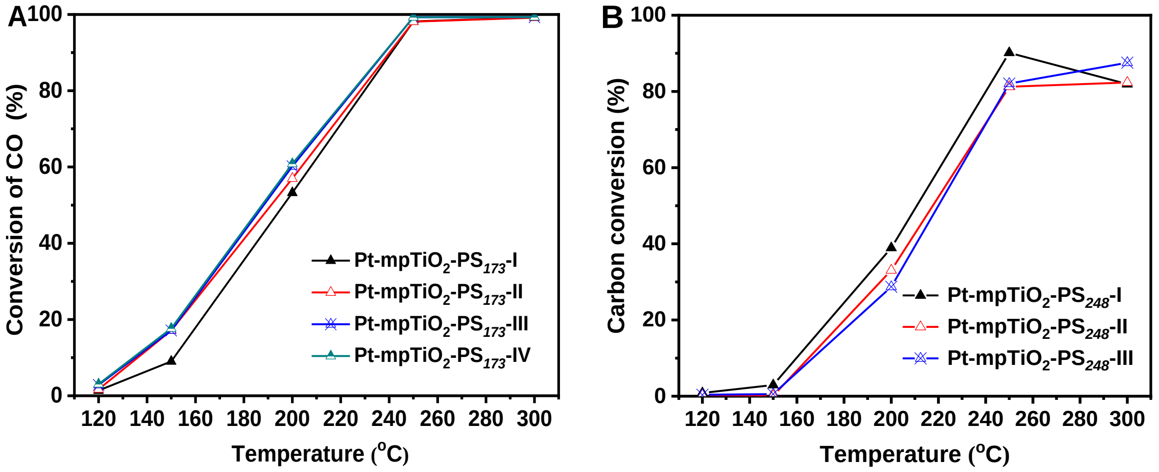

Figure S9. CO conversion curves as a function of temperature over (**A**) Pt-mpTiO_2-PS*173* and (**B**) Pt-mpTiO_2-PS*248* during the cyclic activity evaluation tests. (I-IV refer to the test order during performance evaluation of the recycled catalysts)

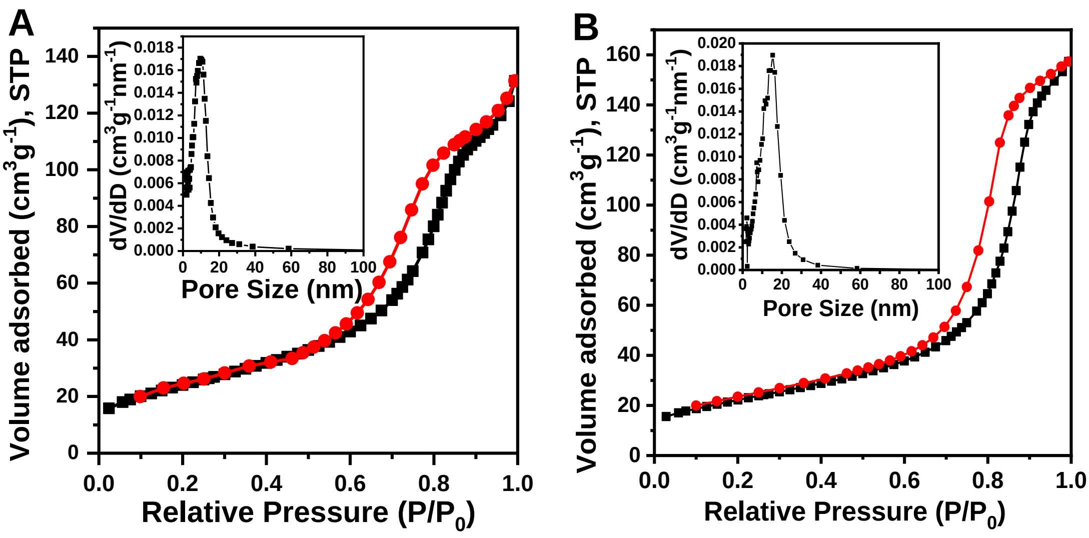

Figure S10. N_2 adsorption-desorption isotherms and pore size distribution curves of the (**A**) used Pt-mpTiO_2-PS$_{120}$ after cyclic catalytic activity evaluation five times, and (**B**) used Pt-mpTiO_2-PS$_{120}$ after long-term catalytic stability evaluation for about 96 h at 250 °C.

N_2 adsorption-desorption isotherms of used Pt-mpTiO_2-PS$_{120}$ catalysts after cyclic catalytic activity evaluation five times and long-term catalytic stability evaluation for 96 h at 250 °C display the typical type-IV isotherms with H_1-type hysteresis loops (Figure S10). Similar isothermal curves and pore size distributions of the used catalysts as that of the fresh one indicates the structure stability of the Pt-mpTiO_2-PS$_{120}$ catalyst.

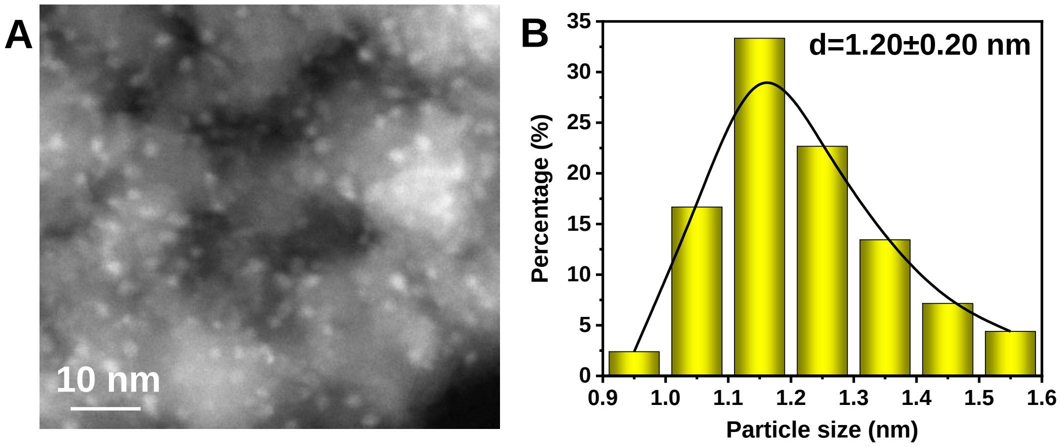

Figure S11. (**A**) HAADF-STEM image and (**B**) particle size distribution of Pt on the used Pt-mpTiO_2-PS$_{120}$ after long-term catalytic stability evaluation at 250 °C obtained according to the HAADF-STEM image.

From Figure S11, it can be found that Pt NCs on the used Pt-mpTiO_2-PS$_{120}$ after long-term test for about 96 h remains the uniform size of ~1.2 nm, which is similar as that of the fresh catalyst, indicating an outstanding stability of the Pt species on the Pt-mpTiO_2-PS$_{120}$ catalyst.

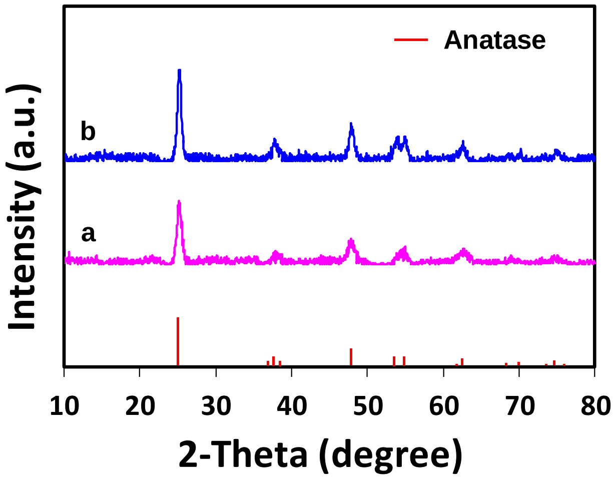

Figure S12. XRD patterns of the (a) used Pt-mpTiO_2-PS$_{120}$ after cyclic activity evaluation five times, (b) used Pt-mpTiO_2-PS$_{120}$ after catalytic stability and cyclic performance evaluation for about 96 h at 250 °C.

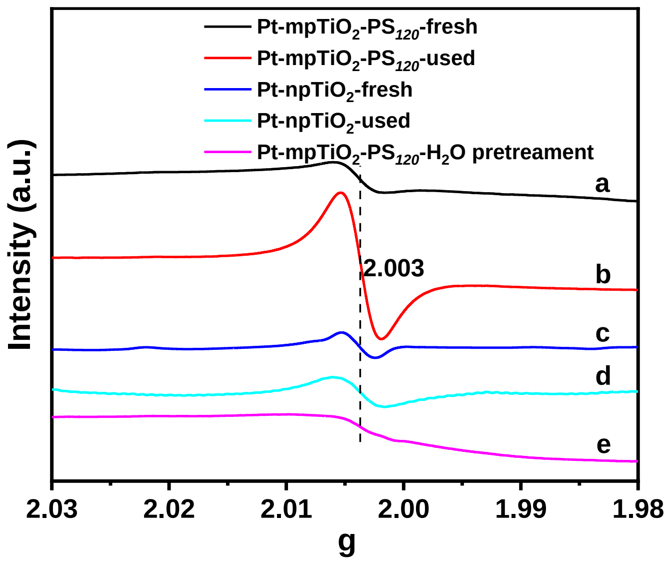

Figure S13. EPR spectra of the (a) fresh Pt-mpTiO_2-PS$_{120}$, (b) used Pt-mpTiO_2-PS$_{120}$, (c) fresh Pt-npTiO_2, (d) used Pt-npTiO_2, and (e) Pt-mpTiO_2-PS$_{120}$ treated by steam for 30 h. The used catalysts refer to those after cyclic activity evaluation five times.

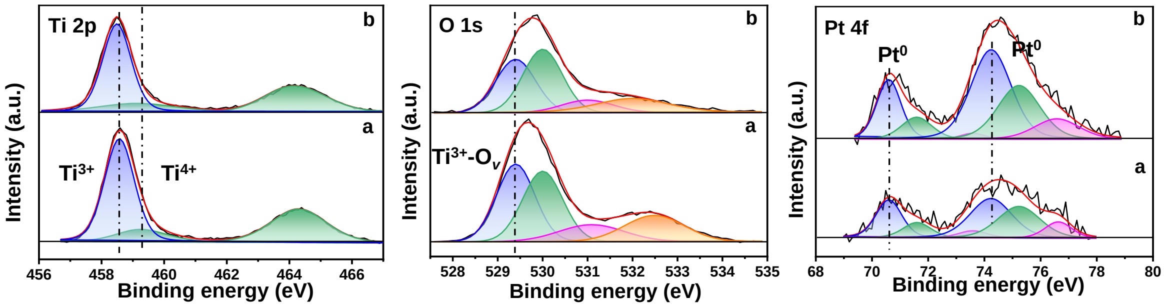

Figure S14. XPS spectra of Pt 4f, Ti 2p and O 1s of the (a) fresh Pt-npTiO_2 and (b) used Pt-npTiO_2 after continuous evaluation of catalytic activity five times.

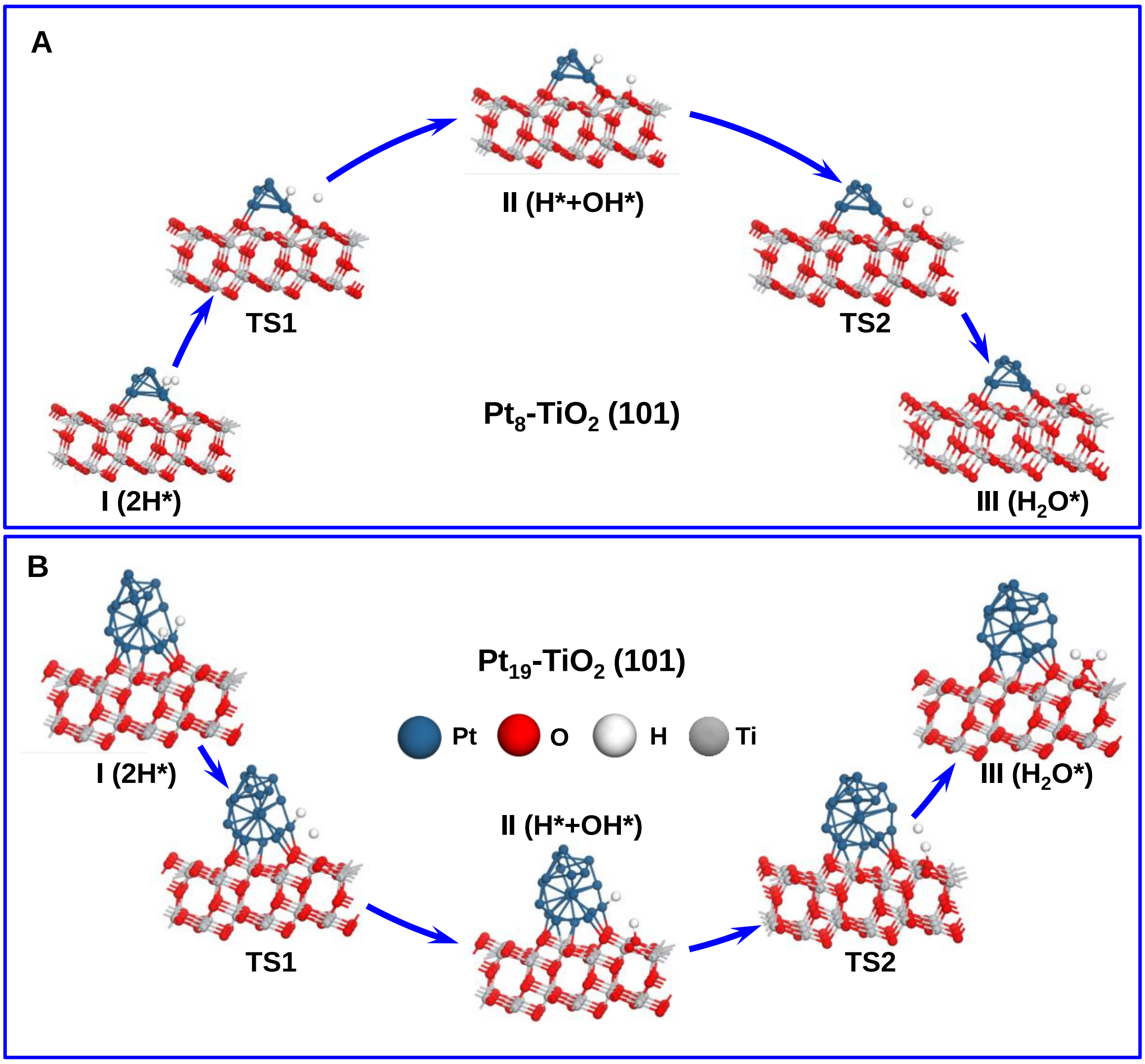

Figure S15. Reaction path for the formation of O_v on (**A**) Pt_8-TiO_2 (101) and (**B**) Pt_{19}-TiO_2 (101) by hydrogen reduction. The structures of intermediates and transition states (TSs) of the key elementary steps are shown in the reaction process. TS1 and TS2 refer to the transfer states of dissociated hydrogen to lattice oxygen.

Density functional theory (DFT) calculations were conducted to reveal the reduction process of different catalysts by hydrogen and the formation mechanism of O_v (Figure S15). In order to distinguish the size of Pt species on the mesoporous and nonporous catalysts, the Pt-mpTiO_2-PS_{120} and Pt-npTiO_2 were simplified and modeled as Pt_8-TiO_2 (101) and Pt_{19}-TiO_2 (101). The starting configuration involves two dissociated H* on the Pt sites (Figure S15 intermediate I). One of the dissociated H* can transfer to the adjacent lattice

oxygen on TiO_2 to active the lattice oxygen (Figure S15 intermediate II), and the other dissociated H* would transfer to the lattice oxygen in succession to remove the oxygen along with the release of a H_2O molecule (Figure S15 intermediate III).

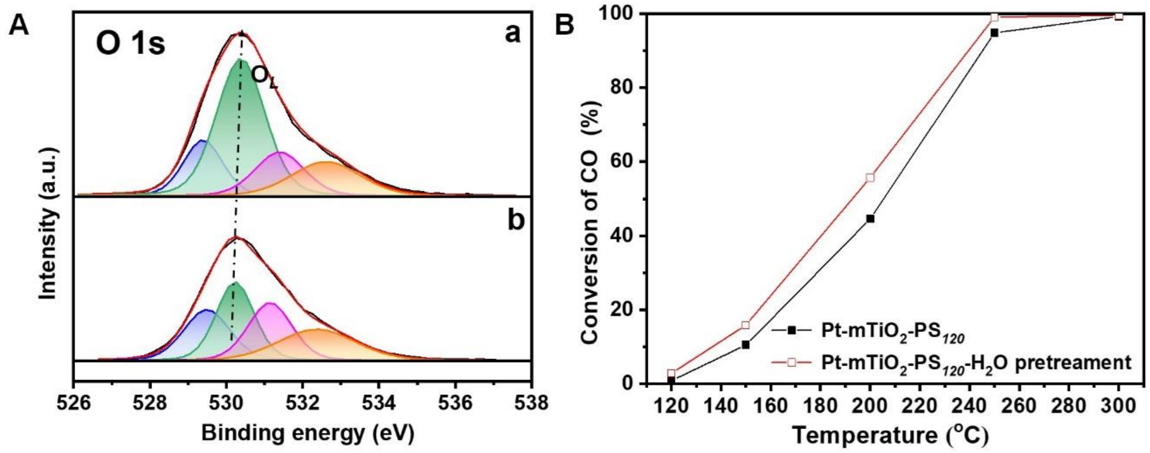

Supplementary Fig. 16. (**A**) O 1s XPS spectra of (a) Pt-mpTiO$_2$-PS$_{120}$ treated by H_2O and (b) fresh Pt-mpTiO$_2$-PS$_{120}$; (**B**) CO conversion curves as a function of temperature for the fresh Pt-mpTiO$_2$-PS$_{120}$ and Pt-mpTiO$_2$-PS$_{120}$ treated by H_2O.

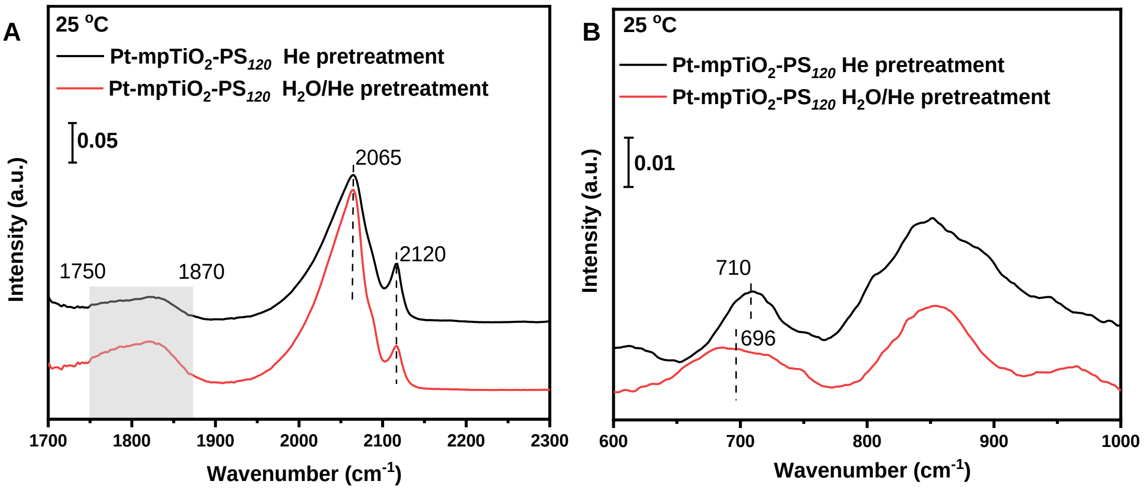

Figure S17. *In situ* DRIFTS spectra of Pt-mpTiO$_2$-PS$_{120}$ recorded in (**A**) 1700-2300 cm^{-1} and (**B**) 600-1000 cm^{-1} after different pretreatment process at 300 °C and CO chemisorption at 25 °C following by subsequent flushing with He for 10 min.

As can be seen from Figure. S17, there is no obvious difference in the bands of CO adsorbed on Pt sites between Pt-mpTiO$_2$-PS$_{120}$ and steam-treated Pt-mpTiO$_2$-PS$_{120}$ (Figure S17A). However, the band of Ti-O (710 cm^{-1}) in the Pt-mpTiO$_2$-PS$_{120}$ displays a red-shift (696 cm^{-1}) and becomes weaker after steam treatment (Figure S17B), indicating the activation of lattice oxygen on TiO$_2$ by hydrogen radical (H*) derived from H_2O dissociation.

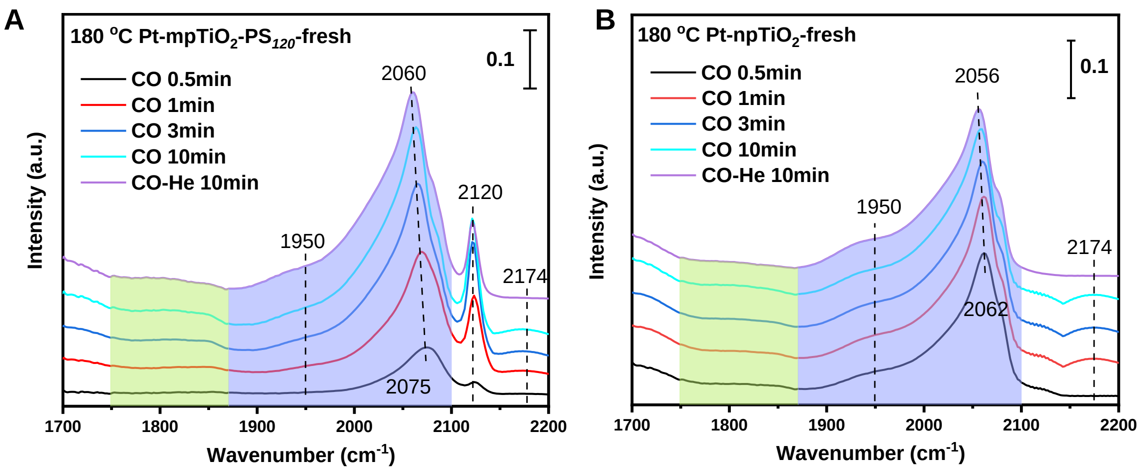

Figure 18. *In situ* DRIFTS spectra of CO chemisorption over fresh (**A**) Pt-mpTiO_2-PS_{120} and (**B**) Pt-npTiO_2 at 180 °C. The catalysts after pretreatment in He atmosphere at 300 °C for 30 min were chemisorbed by CO at 180 °C for 10 min (labeled as CO *x* min, *x* refers to the time of CO adsorption) following by subsequent flushing with He for 10 min (labeled as CO-He 10 min).

Figure S18 shows that, the IR spectra of adsorbed CO between 1870 and 2100 cm^{-1} are ascribed to CO linear adsorption on metallic Pt sites, and the band at ~2120 cm^{-1} is characteristic of CO adsorbed on Pt single atoms, namely isolated ionic Pt sites, while the bands between 1750 and 1870 cm^{-1} correspond to CO bridge adsorption.[4-7]

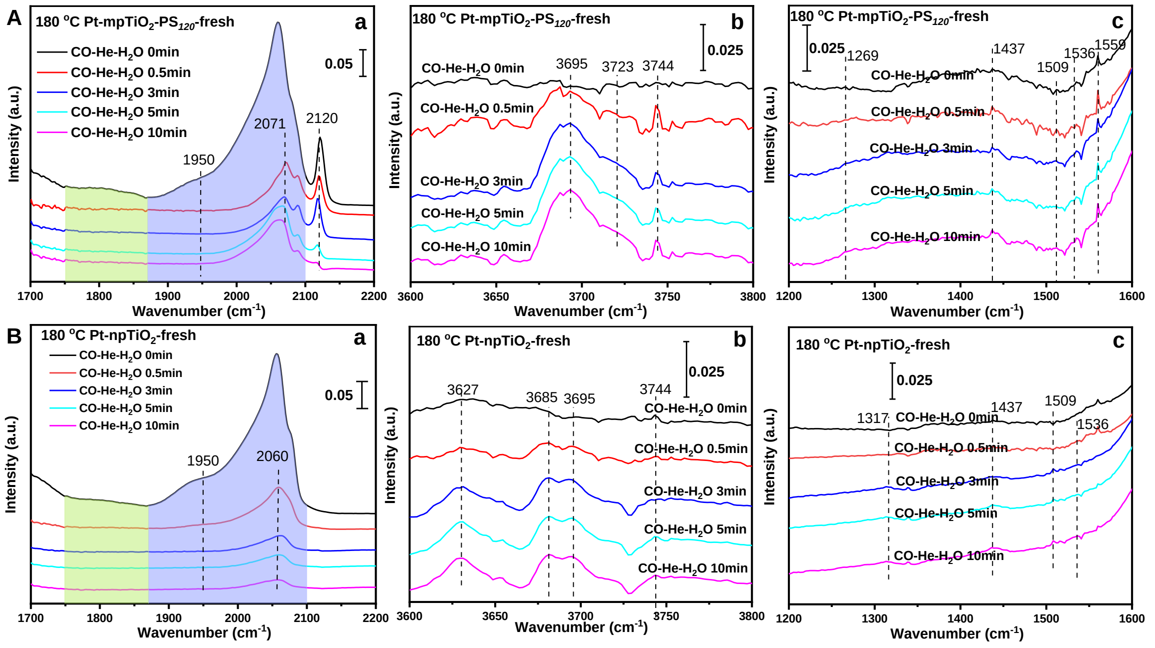

Figure 19. *In situ* time-resolved DRIFTS spectra of CO chemisorption on fresh (**A**) Pt-mpTiO_2-PS*120* and (**B**) Pt-npTiO_2 catalysts recorded in (a) 1700-2200 cm^{-1}, (b) 3600-3800 cm^{-1}, and (c) 1200-1600 cm^{-1} upon exposure to H_2O atmosphere at 180 °C as a function of reaction time (denoted as CO-He-H_2O *x* min, *x* refers to the time of H_2O injection).

For the Pt-mpTiO_2-PS*120*, with the introduction of H_2O at 180 °C, the bands of CO adsorbed on Pt sites decrease gradually (Figure S19Aa) along with the appearance of Ti^{3+}-OH (3723 and 3744 cm^{-1}) and Ti^{4+}-OH (3695 cm^{-1}) bands (Figure S19Ab),[8,9] indicating that the Ti^{3+}-O_v directly participates in H_2O dissociation. The bands at 2320 and 2374 cm^{-1} attributed to CO_2 (Figure S20A) demonstrate that CO activated on Pt sites reacts with H_2O being dissociated at the adjacent O_v to produce CO_2 molecules, and the Ti^{3+}-O_v-$Pt^{\delta+}$ is the active site for WGS reaction. Accordingly, the bands corresponding to the CO_2^- (1269 cm^{-1}), monodentate (m-CO_3^{2-}, 1536 cm^{-1}), bidentate carbonate (b-CO_3^{2-} , 1559 cm^{-1}), and bicarbonate type carbonate species (HCO_3^-, 1437 cm^{-1})[4,8,9] are formed (Figure S19Ac). No bands assigned to the C-H stretching (2800-3000 cm^{-1}) (Figure S20A) of HCOO-[9] and O-H stretching (~3480 cm^{-1}) (Figure S21A) of -COOH are observed, suggesting that the WGS

reaction over the Pt-mpTiO_2-PS_{120} catalyst exclusively undergoes redox pathway rather than associative pathway.

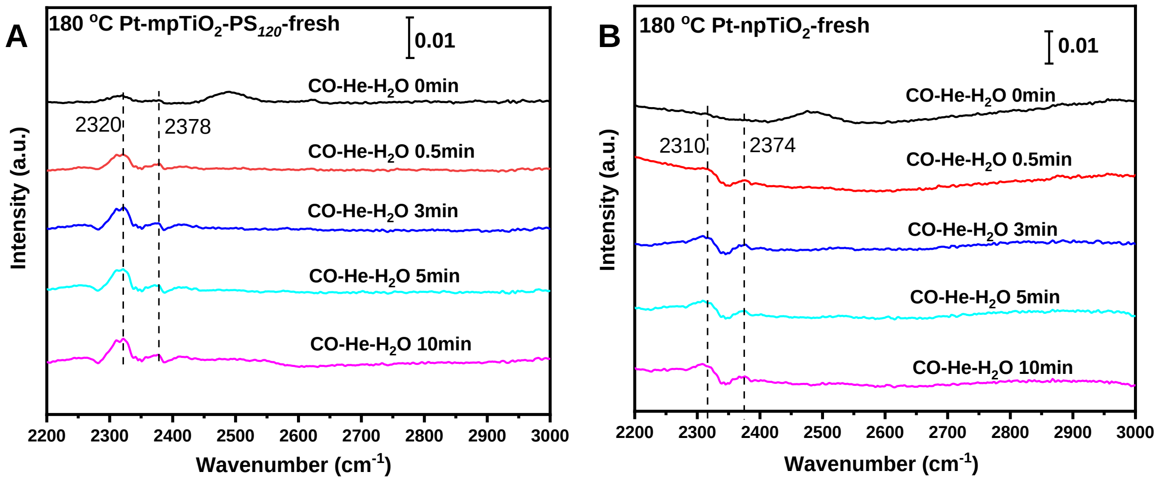

Figure S20. *In situ* time-resolved DRIFTS spectra of CO chemisorption on fresh (**A**) Pt-mpTiO_2-PS$_{120}$ and (**B**) Pt-npTiO_2 recorded in 2200-3000 cm^{-1} upon exposure to H_2O atmosphere at 180 °C as a function of reaction time (denoted as CO-He-H_2O *x* min, *x* refers to the time of H_2O injection).

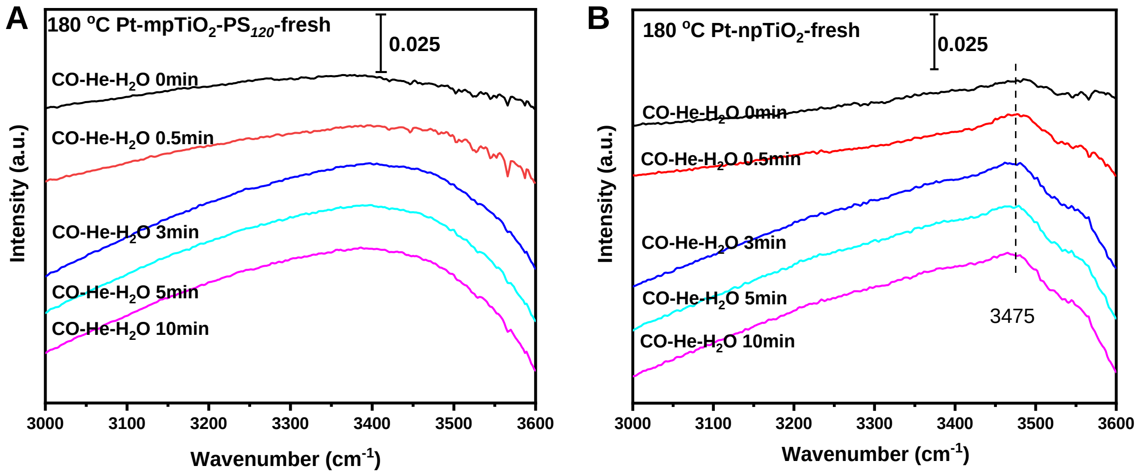

Figure S21. *In situ* time-resolved DRIFTS spectra of CO chemisorption on fresh (**A**) Pt-mpTiO$_2$-PS$_{120}$ and (**B**) Pt-npTiO$_2$ recorded in 3000-3600 cm^{-1} upon exposure to H_2O atmosphere at 180 °C as a function of reaction time (denoted as CO-He-H_2O *x* min, *x* refers to the time of H_2O injection).

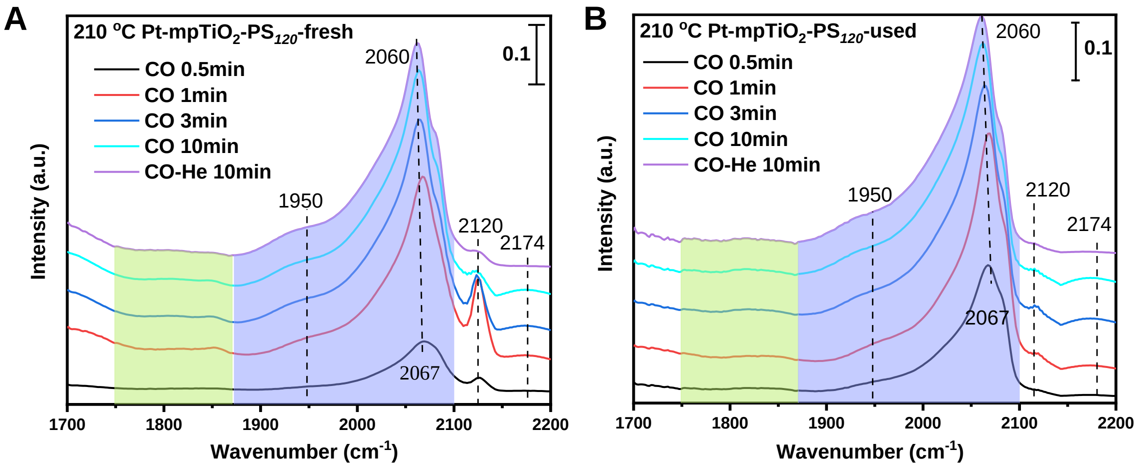

Figure S22. *In situ* DRIFTS spectra of CO chemisorption over (**A**) fresh Pt-mpTiO_2-PS$_{120}$ and (**B**) used Pt-mpTiO_2-PS$_{120}$ after cyclic activity evaluation five times at 210 °C. The catalyst pretreated in He atmosphere at 300 °C for 30 min was chemisorbed by CO at 210 °C for 10 min (labeled as CO *x* min, *x* refers to the time of CO adsorption) following by subsequent flushing with He for 10 min (labeled as CO-He 10 min).

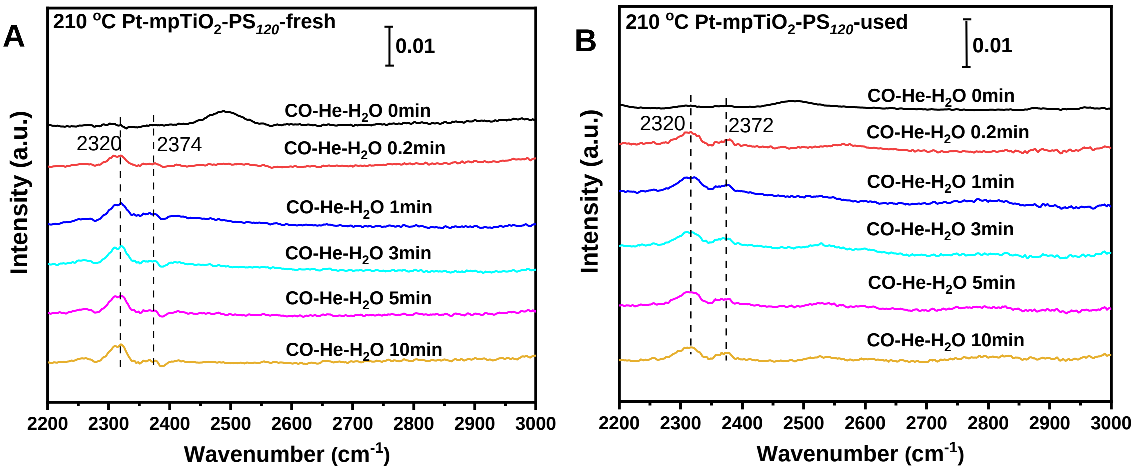

Figure S23. *In situ* time-resolved DRIFTS spectra of CO chemisorption on (**A**) fresh Pt-mpTiO_2-PS*120* and (**B**) used Pt-mpTiO_2-PS*120* after cyclic activity evaluation five times recorded in 2200-3000 cm^{-1} upon exposure to H_2O atmosphere at 210 °C as a function of reaction time (denoted as CO-He-H_2O *x* min, *x* refers to the time of H_2O injection).

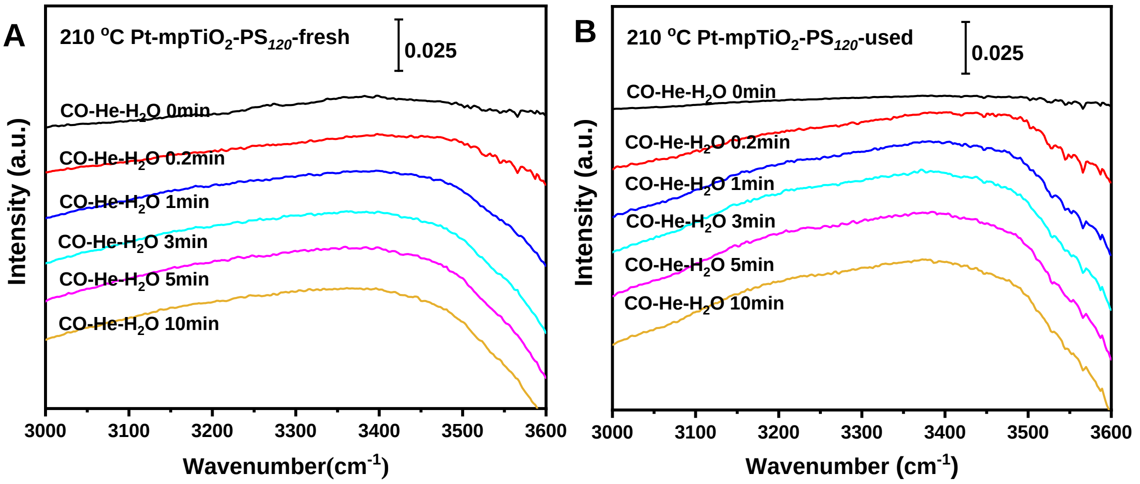

Figure S24. *In situ* time-resolved DRIFTS spectra of CO chemisorption on (**A**) fresh Pt-mpTiO_2-PS*120* and (**B**) used Pt-mpTiO_2-PS*120* after cyclic activity evaluation five times recorded in 3000-3600 cm^{-1} upon exposure to H_2O atmosphere at 210 °C as a function of reaction time (denoted as CO-He-H_2O *x* min, *x* refers to the time of H_2O injection).

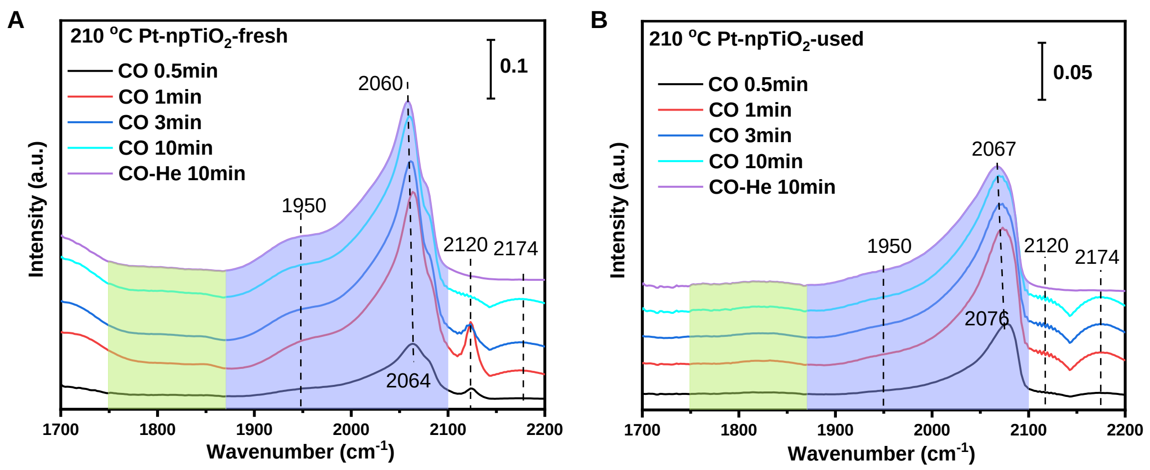

Figure S25. *In situ* DRIFTS spectra of CO chemisorption over (**A**) fresh Pt-npTiO_2 and (**B**) used Pt-npTiO_2 catalysts after cyclic activity evaluation five times at 210 °C. The catalyst after pretreatment in He atmosphere at 300 °C for 30 min was chemisorbed by CO at 210 °C for 10 min (labeled as CO *x* min, *x* refers to the time of CO adsorption) following by subsequent flushing with He for 10 min (labeled as CO-He 10 min).

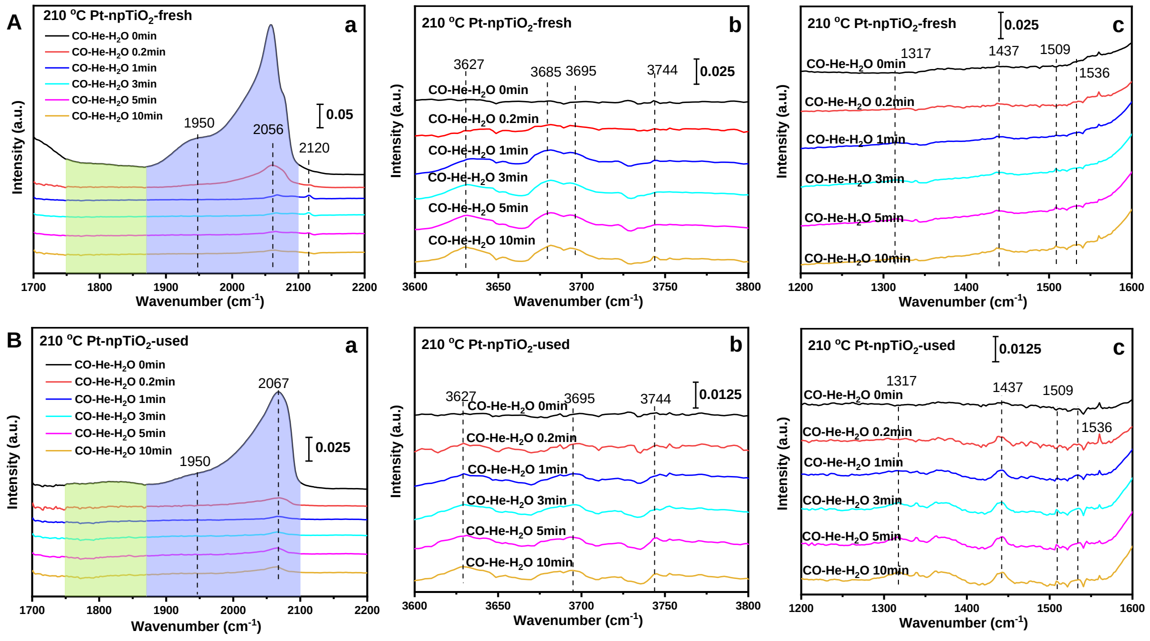

Figure S26. *In situ* time-resolved DRIFTS spectra of CO chemisorption on (**A**) fresh Pt-npTiO$_2$ and (**B**) used Pt-npTiO$_2$ catalysts after cyclic activity evaluation five times recorded in (a) 1700-2200 cm^{-1}, (b) 3600-3800 cm^{-1}, and (c) 1200-1600 cm^{-1} upon exposure to H_2O atmosphere at 210 °C as a function of reaction time (denoted as CO-He-H_2O *x* min, *x* refers to the time of H_2O injection).

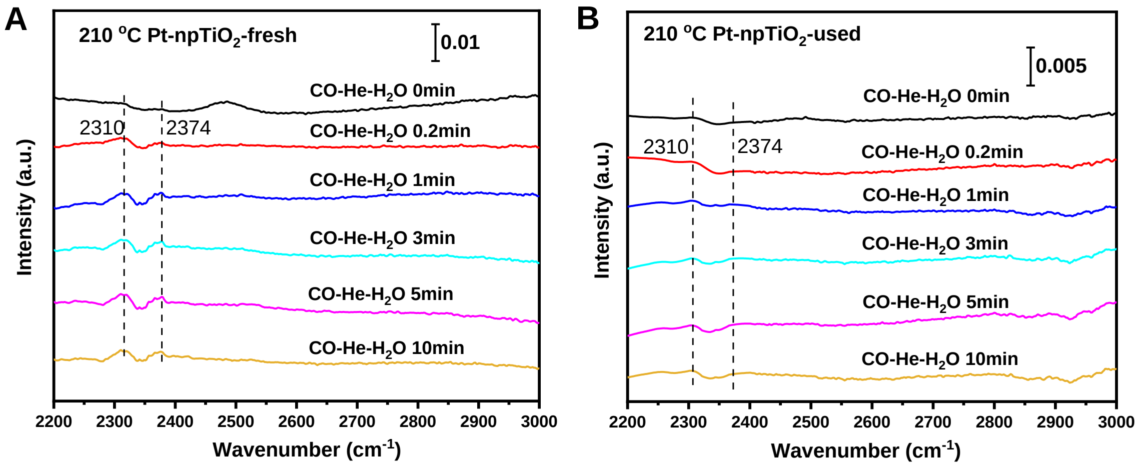

Figure S27. *In situ* time-resolved DRIFTS spectra of CO chemisorption on (**A**) fresh Pt-npTiO$_2$ and (**B**) used Pt-npTiO$_2$ catalysts after cyclic activity evaluation five times recorded in 2200-3000 cm^{-1} upon exposure to H_2O atmosphere at 210 °C as a function of reaction time (denoted as CO-He-H_2O *x* min, *x* refers to the time of H_2O injection).

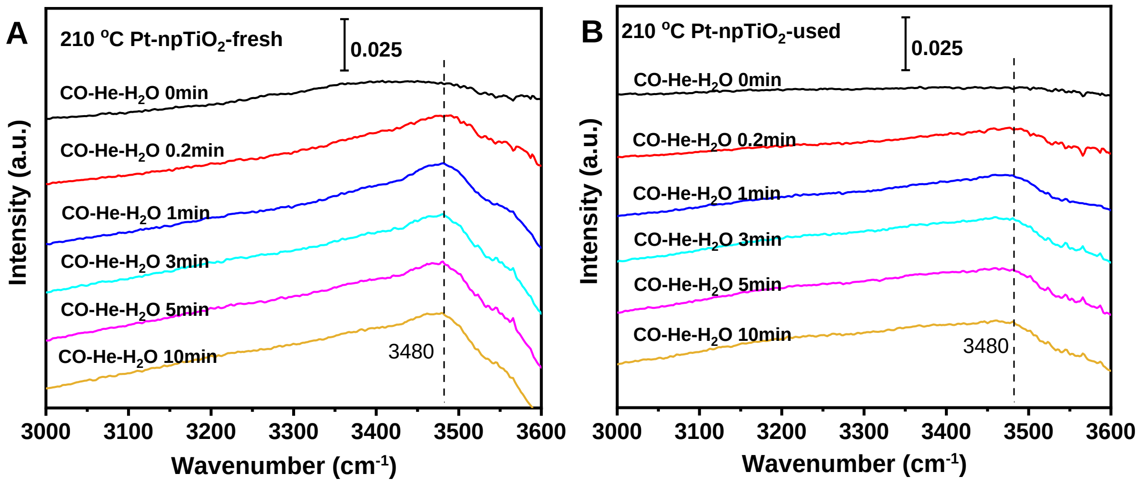

Figure S28. *In situ* time-resolved DRIFTS spectra of CO chemisorption on (**A**) fresh Pt-npTiO$_2$ and (**B**) used Pt-npTiO$_2$ catalysts after cyclic activity evaluation five times recorded in 3000-3600 cm^{-1} upon exposure to H_2O atmosphere at 210 °C as a function of reaction time (denoted as CO-He-H_2O *x* min, *x* refers to the time of H_2O injection).

For Pt-npTiO$_2$, with the introduction of H_2O, the band (~3480 cm^{-1}) corresponding to O-H stretching mode of -COOH was observed in Figure S21B and Figure S28, demonstrating the carboxyl associative pathway of the WGS reaction over Pt-npTiO$_2$.

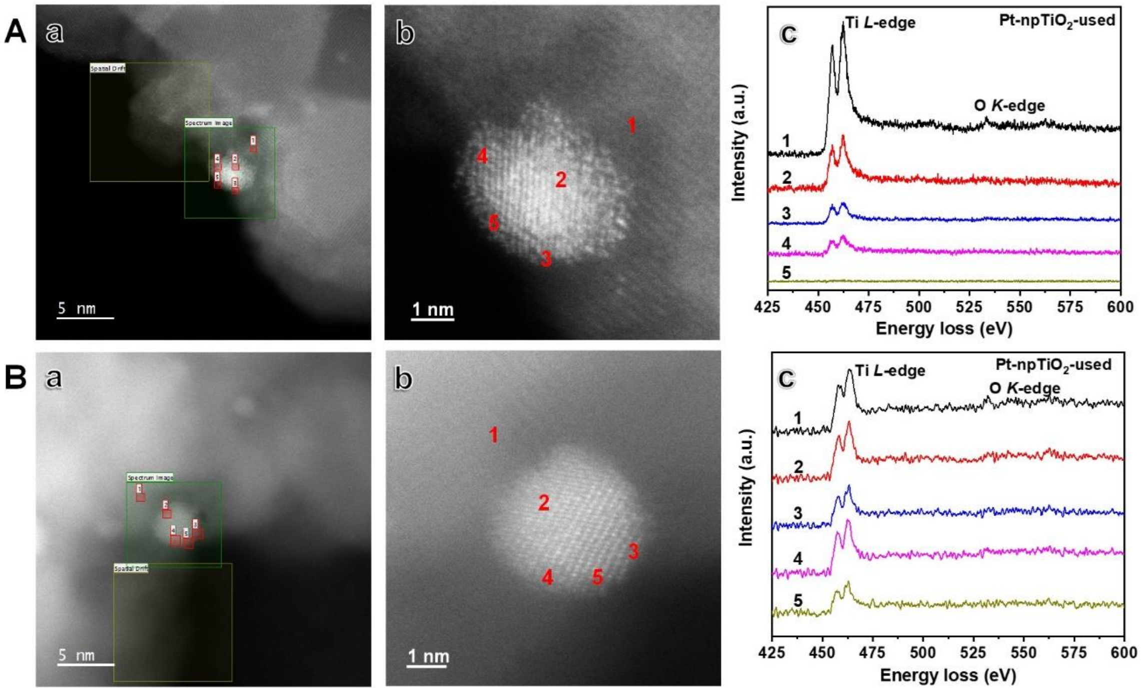

Figure S29. EELS analysis of (**A**) fresh Pt-npTiO_2 and (**B**) used Pt-npTiO_2 catalysts after cyclic activity evaluation five times. (a) Survey image for the EELS analysis; (b) The corresponding Ac-HAADF-STEM image; (c) The EELS spectra of the selected spots (red mark) in (a) and (b).

According to the extracted EELS spectra shown in Figure S29A, it can be seen that the Pt NPs were partially encapsulated by TiO_2 in the fresh Pt-npTiO_2 catalyst, while in the used Pt-npTiO_2 (Figure S29B), the Pt NPs were almost completely encapsulated, indicating the over-encapsulation of large Pt NPs caused by the SMSI during WGS.

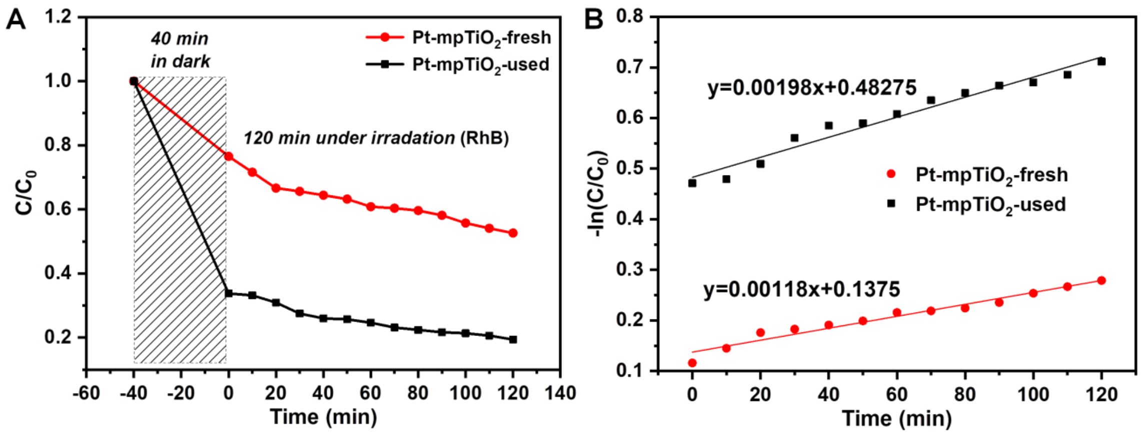

Figure S30. Catalytic performance of fresh and used Pt-mpTiO_2-PS$_{120}$ catalysts towards photocatalytic degradation of Rhodamine B.

From Figure S30, it can be seen that the degradation rate of Rhodamine B over the used Pt-mpTiO_2-PS$_{120}$ is almost twice that over the fresh catalyst, indicating its excellent catalytic performance as a result of enhanced activity.

Table S1. Textural properties of $mpTiO_2$-PS_x and fresh Pt-$mpTiO_2$-PS_x.

Sample	BET surface area (m^2/g)	Pore size (nm)	Window size (nm)	Pore volume (cm^3/g)
$mpTiO_2$-PS_{120}	140	11.2	7.91	0.344
$mpTiO_2$-PS_{173}	113	14.9	10.2	0.349
$mpTiO_2$-PS_{248}	92.3	24.6	15.8	0.365
Pt-$mpTiO_2$-PS_{120}	100	9.59	7.24	0.234
Pt-$mpTiO_2$-PS_{173}	104	12.0	7.96	0.229
Pt-$mpTiO_2$-PS_{248}	76.2	20.8	11.3	0.229

Table S2. Loading amount of Pt on diverse fresh Pt-TiO_2 catalysts.

Sample	Pt (wt%)
Pt-mpTiO_2-PS_{120}	1.72
Pt-mpTiO_2-PS_{173}	1.55
Pt-mpTiO_2-PS_{248}	1.49
Pt-npTiO_2	1.26

Table S3. Relative contents of different composition of various Pt-TiO_2 catalysts.

Sample	Pt 4f	Ti 2p	O 1s
	$Pt^0/(Pt^0+Pt^{2+}+Pt^{4+})$ ratio	$Ti^{3+}/(Ti^{3+}+Ti^{4+})$ ratio	Ti^{3+}-O_v/(Ti^{4+}-O+Ti^{3+}-O_v) ratio
Pt-mpTiO_2-PS_{120}-fresh	0.47	0.48	0.42
Pt-mpTiO_2-PS_{120}-used[a]	0.56	0.64	0.65
Pt-mpTiO_2-PS_{120}-used[b]	0.57	0.58	0.59
Pt-npTiO_2-fresh	0.48	0.54	0.51
Pt-npTiO_2-used	0.57	0.52	0.46

a: after cyclic catalytic activity evaluation five times, *b*: after long-term catalytic stability evaluation at 250 °C. $Pt^0/(Pt^0+Pt^{2+}+Pt^{4+})$ ratio was calculated from Pt 4f XPS spectra. $Ti^{3+}/(Ti^{3+}+Ti^{4+})$ ratio was calculated from Ti 2p XPS spectra. Ti^{3+}-O_v/(Ti^{4+}-O+ Ti^{3+}-O_v) ratio was calculated from O 1s XPS spectra.

Table S4. Comparison of the WGS reaction activities over the representative reducible oxide-supported noble metal catalytic systems.

Catalyst	Temp (°C)	Reactant gas composition	metal normalized activity (mol_{CO} s^{-1} mol_{metal}^{-1})	Ref
Au@TiO_{2-x}/ZnO(H300)	250	6%CO+25%H_2O+Ar	0.15	10
Au clusters-CeO_2	200	2%CO+12%H_2O+N_2	0.10	11
Au particles-CeO_2	200	2%CO+12%H_2O+N_2	0.02	11
Pt-CeO_2/TiO_2	200	4.4%CO+29.6%H_2O+28%H_2+8.7%CO_2+0.1%CH_4+N_2	0.09	12
Pt-CeO_2/Fe_2O_3/Al_2O_3	250	4.5%CO+31.1kPaH_2O+N_2	0.03	13
Pt-CeO_2/ZrO_2	228	5.92%CO+26%H_2O+31.82%H_2+7.4%CO_2+ N_2	0.14	14
2wt%Pt-CeO_2	300	10%CO+20%H_2O+Ar	0.15	15
0.5wt%Pt-TiO_2	200	3%CO+10%H_2O+He	0.09	16
Pt-CeO_2	250	11%CO+26%H_2O+26%H_2+7%CO_2+He	0.17	17
Au-CeO_2	250	11%CO+26%H_2O+26%H_2+7%CO_2+He	0.13	17
2wt%Au-CeO_2	200	11%CO+26%H_2O+26%H_2+7%CO_2+N_2	0.02	18
Pt-mpTiO_2-PS_{120}	200	2%CO+8%H_2O+ N_2	0.27	**This work**
Pt-npTiO_2	200	2%CO+8%H_2O+ N_2	0.04	**This work**

The Pt normalized activity of Pt-mpTiO_2-PS_{120} is 0.27 mol_{CO} mol_{Pt}^{-1} s^{-1} at 200 °C, which is up to 13.5 times higher than previously reported similar catalysts (e.g., Au particles-CeO_2) in Table S4.

Table S5. Apparent activation energies (*Ea*) and pre-exponential factors (*A*) of WGS reaction over different Pt-TiO_2 catalysts.

Sample	*Ea* (KJ/mol)	*A*
Pt-mpTiO_2-PS_{120}	67.5	3.2E8
Pt-mpTiO_2-PS_{173}	69.9	4.7E8
Pt-mpTiO_2-PS_{248}	70.6	3.0E8
Pt-npTiO_2	67.6	4.0E7

Table S6. Structures of furfural and the products generated during furfural hydrogenation.

Sample	Structure
Furfural	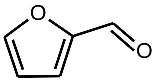
2-MF (target product)	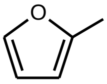
Furan (by product)	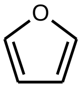
HMF (by product)	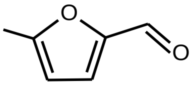
FA (by product)	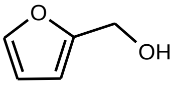
MF-MF (by product)	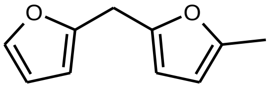
MF=MF (by product)	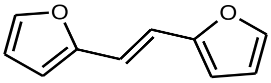
MF-MF-MF (by product)	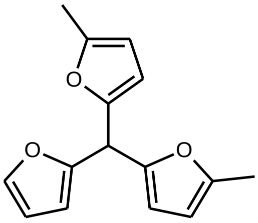
$C_{13}H_{26}$ (by product)	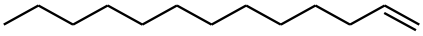
$C_{13}H_{26}O$ (by product)	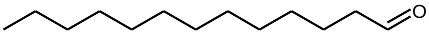
$C_{14}H_{24}O$ (by product)	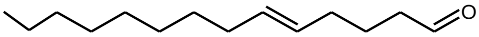
$C_{19}H_{40}O$ (by product)	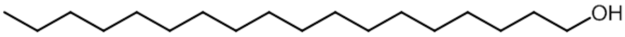

Table S7. Comparison for the activity of furfural hydrogenation to 2-methylfuran (MF) over varied catalysts

Catalyst	Condition	Performance	Ref.
$Cu/Cr-Al_2O_3$<br>$Cu/Cr/Ba/Ca-Al_2O_3$	220 °C, LHSV=0.6 h^{-1},	100% conversion<br>90% selectivity	19
$Cu/Re-Al_2O_3$	220 °C, 4 h,<br>0.26 M furfural/isopropanol	100% conversion<br>94% yield	20
$Cu/Co-Al_2O_3$	220 °C, 40 bar, 5 h,<br>1.75 M furfural/propanol	100% conversion<br>87% yield	21
$Cu/Ni-Al_2O_3$	200 °C, 2 h, 5bar,<br>0.26 M furfural/propanol	100% conversion<br>41% selectivity	22
Cu/Ni-C	230 °C, 40 bar,<br>0.60 M furfural/propanol	91% conversion<br>48% selectivity	23
$Ni/Fe-SiO_2$	250 °C, 1 atm, WHSV=0.1 h^{-1},<br>H_2/furfural ratio=25	96.3% conversion<br>39.1% yield	24
Cu/Al/Co alloys	240 °C, H_2 60 sccm,<br>furfural 0.006 mol h^{-1}	98.2% conversion<br>66.0% selectivity	25
Mg/Fe/O	300 °C, 1 h,<br>5%furfural/45% methanol	57% conversion<br>54% selectivity	26
Ir-C (2% Ir)	220 °C, 5 h, 100 psi,<br>0.27 M furfural/isopropanol	94% conversion<br>70% selectivity	27
Pt-C<br>Ru-C<br>Ni-C	240 °C, 40 bar, 2 h,<br>0.60 M furfural/propanol	50% selectivity for 3%Pt<br>49% for 3%Ru<br>37% for 10%Ni	28
$Ru-NiFe_2O_4$	180 °C, 2.1 MPa, 2 h,<br>0.43 M furfural/propanol	97% conversion<br>83% yield	29
Co/Pt-C	180 °C, 0.5 Mpa, 8 h,<br>0.10 M furfural/isopropanol	100% conversion<br>59% yield	30
$Cu/Pd-TiO_2$	220 °C, 4 h,<br>0.07 M furfural/isopropanol	100% conversion<br>61.2% selectivity	31
$Pt-mpTiO_2$ (1.72% Pt)	300 °C, LHSV=0.83 h^{-1}	100% conversion<br>90% selectivity	This work

To pursue the comparable catalytic performance of furfural selective hydrogenation to 2-MF as that of the industrial catalyst $Cu/Cr-Al_2O_3$, most Cr-free bimetallic catalysts,

such as Cu/Re-Al_2O_3, Cu/Co-Al_2O_3, Cu/Ni-Al_2O_3, were developed (Table S7). To improve the 2-MF selectivity that is relatively low over supported single noble metal catalysts such as Pt-C, Ru-C, Ir-C, some transition metals and noble metals were also coupled to construct catalysts such as Pt/Co-C and Cu/Pd-TiO_2 (Table S7). In any case, bimetallic catalytic active sites seem to be essential for the high MF selectivity *via* the synergistic catalysis. Notably, in our work, the distinctive role of mpTiO_2 gives the stable and excellent catalytic performance after supporting Pt nanoclusters, thereby achieving the considerable MF selectivity (>90%) superior to most reported bimetallic catalysts.

Supplementary References:

[1] Ma, J.; Ren, Y.; Zhou, X.; Liu, L.; Zhu, Y.; Cheng, X.; Xu, P.; Li, X.; Deng, Y.; Zhao, D. Pt nanoparticles sensitized ordered mesoporous WO_3 semiconductor: Gas sensing performance and mechanism study. *Adv. Funct. Mater.* **2018**, *28*, 1870040.

[2] Zou, Y.; Xi, S.; Bo, T.; Zhou, X.; Ma, J.; Yang, X.; Diao, C.; Deng, Y. Mesoporous amorphous Al_2O_3/crystalline WO_3 heterophase hybrids for electrocatalysis and gas sensing applications. *J. Mater. Chem. A* **2019**, *7*, 21874-21883.

[3] Wang, Q.; He, L.; Zhao, L.; Liu, R.; Zhang, W.; Lu, A. Surface charge-driven nanoengineering of monodisperse carbon nanospheres with tunable surface roughness. *Adv. Funct. Mater.* **2020**, *30*, 1906117.

[4] Kalamaras, C. M.; Panagiotopoulou, P.; Kondarides, D. I.; Efstathiou, A. M. Kinetic and mechanistic studies of the water-gas shift reaction on Pt/TiO_2 catalyst. *J. Catal.* **2009**, *264*, 117-129.

[5] Panagiotopoulou, P.; Papavasiliou, J.; Avgouropoulos, G.; Ioannides, T.; Kondarides, D. I. Water-gas shift activity of doped Pt/CeO_2 catalysts. *Chem. Eng. J.* **2007**, *134*, 16-22.

[6] Ding, K.; Gulec, A.; Johnson, A. M.; Schweitzer, N. M.; Stucky, G. D.; Marks, L. D.; Stair, P. C. Identification of active sites in CO oxidation and water-gas shift over supported Pt catalysts. *Science* **2015**, *350*, 189-192.

[7] Jones, J.; Xiong, H.; DeLaRiva, A. T.; Peterson, E. J.; Pham, H.; Challa, S. R.; Qi, G.; Oh, S.; Wiebenga, M. H.; Hernández, X. I. P.; Wang, Y.; Datye, A. K. Thermally stable single-atom platinum-on-ceria catalysts via atom trapping. *Science* **2016**, *353*, 150-154.

[8] Liu, L. J.; Zhao, C. Y.; Li, Y. Spontaneous dissociation of CO_2 to CO on defective surface of Cu(I)/TiO_{2-x} nanoparticles at room temperature. *J. Phys. Chem. C* **2012**, *116*, 7904-7912.

[9] Xu, M.; Yao, S.; Rao, D.; Niu, Y.; Liu, N.; Peng, M.; Zhai, P.; Man, Y.; Zheng, L.; Wang, B.; Zhang, B.; Ma, D.; Wei, M. Insights into interfacial synergistic catalysis over Ni@TiO_{2-x} catalyst toward water-gas shift reaction. *J. Am. Chem. Soc.* **2018**, *140*, 11241-11251.

[10] Liu, N.; Xu, M.; Yang, Y.; Zhang, S.; Zhang, J.; Wang, W.; Zheng, L.; Hong, S.; Wei, M. $Au^{\delta-}$-O_v-Ti^{3+} interfacial site: Catalytic active center toward low-temperature water gas shift reaction. *ACS Catal.* **2019**, *9*, 2707-2717.

[11] Fu, X.; Guo, L.; Wang, W.; Ma, C.; Jia, C.; Wu, K.; Si, R.; Sun, L.; Yan, C. Direct identification of active surface species for the water-gas shift reaction on a gold-ceria catalyst. *J. Am. Chem. Soc.* **2019**, *141*, 4613-4623.

[12] González, I. D.; Navarro, R. M.; Wen, W.; Marinkovic, N.; Rodriguéz, J. A.; Rosa, F.; Fierro, J. L. G. A comparative study of the water gas shift reaction over platinum catalysts supported on CeO_2, TiO_2 and Ce-modified TiO_2. *Catal. Today* **2010**, *149*, 372-379.

[13] Gonzalez Castaño, M.; Reina, T. R.; Ivanova, S.; Centeno, M. A.; Odriozola, J. A. Pt vs. Au in water-gas shift reaction. *J. Catal.* **2014**, *314*, 1-9.

[14] Ruettinger, W.; Liu, X.; Farrauto, R. J. Mechanism of aging for a Pt/CeO_2-ZrO_2 water gas shift catalyst. *Appl. Catal. B Environ.* **2006**, *65*, 135-141.

[15] Thinon, O.; Diehl, F.; Avenier, P.; Schuurman, Y. Screening of bifunctional water-gas shift catalysts. *Catal. Today* **2008**, *137*, 29-35.

[16] Panagiotopoulou, P.; Kondarides, D. I. Effect of morphological characteristics of TiO_2-supported noble metal catalysts on their activity for the water-gas shift reaction. *J. Catal.* **2004**, *225*, 327-336.

[17] Fu, Q.; Saltsburg, H.; Flytzani-Stephanopoulos, M. Active nonmetallic Au and Pt species on ceria-based water-gas shift catalysts. *Science* **2003**, *301*, 935-938.

[18] Yao, S.; Zhang, X.; Zhou, W.; Gao, R.; Xu, W.; Ye, Y.; Lin, L.; Wen, X.; Liu, P.; Chen, B.; Crumlin, E.; Guo, J.; Zuo, Z.; Li, W.; Xie, J.; Lu, L.; Kiely, C. J.; Gu, L.; Shi, C.; Rodrigue, J. A.; Ma, D.

Atomic-layered Au clusters on α-MoC as catalysts for the low-temperature water-gas shift reaction. *Science* **2017**, *357*, 359-393.

[19] Li, Z. X.; Han, T. Z.; Guo, W. X.; Wang, G. M. The comparison of different preparation methods of catalysts for furfural hydrogenization to 2-methylfuran. *Adv. Mater. Res.* **2013**, *791-793*, 68-71.

[20] Dohade, M. G.; Dhepe, P. L. One pot conversion of furfural to 2-methylfuran in the presence of PtCo bimetallic catalyst. *Clean Techn. Environ. Policy*. **2018**, *20*, 703-713.

[21] Srivastava, S.; Jadeja, G. C.; Parikh, J. Copper-cobalt catalyzed liquid phase hydrogenation of furfural to 2-methylfuran: An optimization, kinetics and reaction mechanism study. *Chem. Eng. Res. Des*. **2018**, *132*, 313-324.

[22] Kalong, M.; Hongmanorom, P.; Ratchahat, S.; Koo-amornpattana, W.; Faungnawakij, K.; Assabumrungrat, S.; Srifa, A.; Kawi, S. Hydrogen-free hydrogenation of furfural to furfuryl alcohol and 2-methylfuran over Ni and Co-promoted Cu/γ-Al_2O_3 catalysts. *Fuel Process. Technol.* **2021**, *214*, 106721.

[23] Varila, T.; Mäkelä, E.; Kupila, R.; Romar, H.; Hu, T.; Karinen, R.; Puurunen, R. L.; Lassi, U. Conversion of furfural to 2-methylfuran over CuNi catalysts supported on biobased carbon foams. *Catal. Today*. **2021**, *367*, 16-27.

[24] Sitthisa, S.; An, W.; Resasco, D. E. Selective conversion of furfural to methylfuran over silica-supported Ni-Fe bimetallic catalysts. *J. Catal.* **2011**, *284*, 90-101.

[25] Zhou, K.; Chen, J.; Cheng, Y.; Chen, Z.; Kang, S.; Cai, Z.; Xu, Y.; Wei, J. Enhanced catalytic transfer hydrogenation of biomass-based furfural into 2-methylfuran over multifunctional Cu-Re bimetallic catalysts. *ACS Sustainable Chem. Eng.* **2020**, *8*, 16624-16636.

[26] Grazia, L.; Lolli, A.; Folco, F.; Zhang, Y.; Albonetti, S.; Cavani, F. Gas-phase cascade upgrading of furfural to 2-methylfuran using methanol as a H-transfer reactant and MgO based catalysts. *Catal. Sci. Technol*. **2016**, *6*, 4418-4427.

[27] Date, N. S.; Hengne, A. M.; Huang, K. W.; Chikate, R. C.; Rode, C. V. Single pot selective hydrogenation of furfural to 2-methylfuran over carbon supported iridium catalyst. *Green Chem*. **2018**, *20*, 2027-2037.

[28] Mäkelä, E.; Lahti, R.; Jaatinen, S.; Romar, H.; Hu, T.; Puurunen, R. L.; Lassi, U.; Karinen, R. Study of Ni, Pt, and Ru catalysts on wood-based activated carbon supports and their activity in furfural

conversion to 2-methylfuran. *ChemCatChem.* **2018**, *10*, 3269-3283.

[29] Wang, B. W.; Li, C.; He, B.; Qi, J.; Liang, C. H. Highly stable and selective Ru/$NiFe_2O_4$ catalysts for transfer hydrogenation of biomass-derived furfural to 2-methylfuran. *J. Energy Chem.* **2017**, *26*, 799-807.

[30] Hutchings, G. Nanoporous Cu-Al-Co alloys for selective furfural hydrodeoxygenation to 2-methylfuran. *Ind. Eng. Chem. Res.* **2017**, *56*, 3866-3872.

[31] Chang, X.; Liu, A.; Cai, B.; Luo, J.; Pan, H.; Huang, Y. Catalytic transfer hydrogenation of furfural to 2-methylfuran and 2-methyltetrahydrofuran over bimetallic copper-palladium catalysts. *ChemSusChem*. **2016**, *9*, 3330-3337.
